# Pathogenic rickettsiae encode a secreted lipase that facilitates intracytosolic colonization in host cells

**DOI:** 10.1371/journal.pone.0332810

**Published:** 2025-10-08

**Authors:** Mohammad Sadik, Imran Moin, Saif Ullah, Andrew C. Krusenstjerna, Mathilde Gonin, Erin D. Goley, M. Sayeedur Rahman, Oliver H. Voss

**Affiliations:** 1 Department of Microbiology and Immunology, University of Maryland School of Medicine, Baltimore, Maryland, United States of America; 2 Department of Molecular Biology and Genetics, The Johns Hopkins University School of Medicine, Baltimore, Maryland, United States of America; University of Minnesota, UNITED STATES OF AMERICA

## Abstract

Key cellular processes for the rickettsial obligate intracellular lifestyle, including internalization by phagocytosis, regulation of intracellular trafficking, and evasion of lysosomal destruction to establish an intracytosolic replication niche, remain poorly defined. Recent reports showed that rickettsial phospholipases play an important role in vacuolar escape, but their functions are dispensable depending on the host cell-type. Here, we report the identification of a putative lipase (locus_tag: A1G_01170) with a Serine hydrolase motif (GXSXG) in the *R. rickettsii* (Sheila Smith) genome, which we named RLip (*Rickettsia* Lipase). Sequence comparison shows that the Serine hydrolase motif is conserved among RLip molecules of other *Rickettsia* species. Our work reveals that RLip harbors a lipase activity, and its recombinant expression is cytotoxic to yeast and mammalian cells. We further demonstrate that RLip expression is induced during *R. rickettsii* or *R. parkeri* infection, while its expression was minimally detected during *R. montanensis* (non-pathogenic) infection. Fractionation of *R. rickettsii*-infected host cells into cytosolic (carrying secreted proteins) and insoluble pellet (carrying rickettsiae) fractions, shows the presence of RLip in the cytoplasmic fraction, while being minimally retained by the bacteria. Infection studies in HMEC-1 cells using *R. parkeri* wild-type (WT) or *R. parkeri rlip*::Tn (non-functional RLip), demonstrate that lack of RLip function significantly impairs rickettsial evasion from bactericidal phagolysosomal fusion, suggesting that RLip plays a critical role in the escape from membrane-bound vacuoles to facilitate the intracytosolic colonization of pathogenic *Rickettsia* species*.*

## Introduction

Rickettsioses are vector-borne diseases, presenting a perilous threat to public health. In fact, tick- and flea-borne rickettsial diseases are on the rise globally and our inadequate understanding of how *Rickettsia* interacts with their mammalian host has impaired the development of effective interventions against rickettsial disease [1].

The members of the genus *Rickettsia* are obligate intracellular Gram-negative bacteria that infect humans, through the bite, or by the feces, of an infected arthropod vector [2]. During infection, rickettsiae seize control over the host cellular processes, including membrane dynamics, actin cytoskeleton, phosphoinositide (PI) metabolism, intracellular signaling and immune defense responses, to establish a replication niche in the metabolite-rich host cytosol and ultimately disseminate into neighboring cells/organs of the host [3–7]. To orchestrate such a complex cellular tasks *Rickettsia* and other bacteria utilize an arsenal of effectors, including membranolytic enzymes. For instance, *Listeria monocytogenes* uses the cholesterol-dependent cytolysin listeriolysin O (LLO) and several phospholipase C enzymes to promote vacuolar escape [8–12], while *Pseudomonas aeruginosa* releases ExoU, a phospholipase A_2_ (PLA_2_), to complete their pathogenic life cycle [13,14]. In addition, *Legionella pneumophila* secrets two phospholipases, VipD and VpdC, to facilitate their intracellular lifestyle [15,16], while *Shigella flexneri* utilizes the IpaB and IpaC invasins to promote membrane rupture and host invasion [17–19]. In the case of rickettsiae, we reported that rickettsial phospholipase A_2_ enzymes Pat1 (present in all rickettsiae), and Pat2 (variably present) were involved in the phagosomal escape to support host colonization [20–22]. Also, reports from other laboratories revealed that phospholipase D (PLD) (present in all rickettsiae) [23] and Pat1 [24] play a role in vacuolar escape. However, these enzymes were also reported to be dispensable depending on the host cell type [23–25]. Given these reports, we hypothesize that *Rickettsia* possess additional membranolytic effector(s) to regulate the escape from vacuolar membranes, to avoid trafficking to lysosomes and to gain access to the nutrient-rich host cytosol. In this effort, our bioinformatic analysis of the rickettsial genomes [26] resulted in the identification of a putative lipase, which we named RLip (*Rickettsia* Lipase), that harbored a conserved Serine hydrolase motif (GXSXG). Functional characterization revealed that RLip of *R. rickettsii* possesses lipase activity, and its recombinant expression was cytotoxic to yeast and mammalian cells. Infection studies employing either an antibody-mediated neutralization approach of RLip in *R. rickettsii* or using a *R. parkeri rlip*::Tn (non-functional RLip) mutant, resulted in a defect in rickettsial escape from bactericidal phagolysosomal fusion, suggesting that RLip plays a critical role in facilitating the release from vacuolar membranes to contribute to the intracellular survival of pathogenic rickettsiae.

## Materials and methods

### Antibodies and reagents

Anti-LAMP2 (H4B4), anti-GAPDH (FL-335), and horseradish peroxidase (HRP)-conjugated secondary Abs (mouse, rabbit, rat, guinea pig, and goat IgGs) were purchased from Santa Cruz Biotechnology. ProLong Gold antifade mounting medium with DAPI (4′,6-diamidino-2-phenylindole), paraformaldehyde (PFA), Halt protease and phosphatase inhibitor cocktail, HisPur^TM^ Ni-NTA magnetic beads, anti-V5 (SV5-Pk1), anti-His (C-terminal, 46–0693) antibodies (Abs), and Alexa Fluor 488/594-conjugated secondary Abs were purchased from Thermo Fisher Scientific. Anti-Flag (M2) Ab was acquired from Sigma, while the PolyJet transfection reagent was obtained from Signagen. The rabbit Ab against recombinant full-length RLip protein (anti-RLip), encoded by codon-optimized A1G_01170 (locus_tag) of *R. rickettsii* (Sheila Smith) strain, was generated and affinity purified by Thermo Fisher Scientific and specificity was validated by western blot analysis (please see S3 Fig). The rabbit Ab against recombinant Pat1 protein [283 amino acids of the C-terminal region), encoded by locus_tag: A1G_05085] (anti-Pat1) of *R. rickettsii* (Sheila Smith) strain, was generated and affinity purified by Thermo Fisher Scientific. The mouse monoclonal anti-OmpA/B Ab (clone: RC-5H2) against SFG rickettsiae was purchased from Fuller laboratories.

### Bacterial strains, cell culture, and infection

Vero76 (African green monkey kidney, RL-1587; ATCC), SVEC4−10 (CRL-2181, ATCC), and HeLa (CCL-2; ATCC) cells were maintained in minimal Dulbecco’s modified Eagle’s medium (DMEM) supplemented with 10% heat-inactivated fetal bovine serum (FBS) at 37°C with 5% CO_2_. HMEC-1 cells (human microvascular endothelial cells, CDC, Lot-No.: 119223) were grown in HMEC-1 media [MCDB 131 media (Invitrogen) supplemented with 10% FBS (GeminiBio), 10 mM L-glutamine (Gibco), 10 ng/ml epidermal growth factor (Becton-Dickinson), 1 μg/mL hydrocortisone (Sigma), and 1.18 mg/mL sodium bicarbonate]. *R. rickettsii* (Shelia Smith) and *R. montanensis* were obtained from Dr. Ted Hackstadt (Rocky Mountain Laboratories, NIH, MT, USA). All *Rickettsia* species were propagated in Vero76 cells grown in DMEM medium supplemented with 5% FBS at 34°C and 5% CO_2_ and for *R. parkeri rlip*::Tn*,* spectinomycin (50 μg/mL) was added in the culture medium for selection. Rickettsiae from infected host cells were partially purified as described previously [27–29]. Briefly, rickettsiae infected cells were disrupted by vortexing with 1 mm glass beads. The disrupted host cells were centrifuged at 250 x *g* for 5 min at 4°C to remove host cell debris or any remaining intact host cells. The supernatant was centrifuged at 9,000 x *g* for 3 min at 4°C. The pellet containing partially purified rickettsiae were collected for host cell infection. For early stages of infection [before the doubling time (8–10 hrs) of rickettsiae], a higher multiplicity of infection (MOI) of 20 [*e.g.*, 2 hrs post-infection (hpi)] was used to ensure the presence of sufficient number of bacteria, as compared to MOI of 5 at later time points (e.g., 24 hpi), to determine the biological functions of the bacteria during host infection [20,28,30–32].

For the RLip induction experiment, *R. rickettsii* was partially purified using the glass bead method as mentioned above [29]. The partially purified bacteria, resuspended in DMEM media supplemented with 5% FBS, were used to infect Vero76 cells. The rickettsiae-infected host cells were harvested at various post infection times, in ice-cold 1 x PBS supplemented with protease and phosphatase inhibitor cocktails. The harvested cell pellets were lysed by sonication and immunoblotted with anti-RLip, anti-Pat1, anti-OmpA/B, and anti-GAPDH Abs.

### Bioinformatic analysis and homology modelling of RLip

The sequence analyses of RLip using blastp (against the NCBI Conserved Domains Database) [33] and Phyre2 [26] suggests that RLip possesses a secreted lipase with Serine hydrolase motif (GXSXG). The putative active site region of RLip (locus_tag: A1G_01170) from *R. rickettsii* (Sheila Smith) strain was aligned with other bacterial lipases [*Pseudomonas aeruginosa* ExoU (WP_003134060); *Legionella pneumophila* VipD (WP_010948518); *Legionella pneumophila* VpdC (WP_010947155); *R. typhi* Pat1 (WP_011191036), and *R. typhi* Pat2 (AAU03991)]. The sequence alignment of RLip homologs across other *Rickettsia* species [*R. bellii* (WP_012151906); *R. akari* (ABV74559); *R. australis* (WP_014413153); *R. felis* (WP_041405363); *R. montanensis* (WP_014409937); *R. conorii* (WP_010976872); *R. rickettsii* (WP_012150422); *R. parkeri* (WP_014410398); *R. canadensis* (WP_012148317); *R. typhi* (WP_011190628); and *R. prowazekii* (WP_004598629)] was done by Clustal Omega using default parameters. The homology modelling of RLip was performed by automated sever Phyre2 [26]. The model was further validated by Ramachandran plot using WinCoot 0.9.8.95 EL [34] and visualized by PyMOL (Molecular Graphics System, Schrödinger, LLC) (S2 Fig). Continuing analysis of the RLip protein sequence using the web-based SignalP-6.0 program [35] showed no presence of a signal peptide sequence required for Sec translocon.

### Mammalian expression plasmids

The codon-optimized full-length RLip-WT and RLip-S138A mutant were sub-cloned into green fluorescent protein (GFP)-tagged (pcDNA6.2), and FLAG-tagged (pcDNA4/TO/StrepII) plasmid.

### Secretion assay

Monolayer of Vero76, or HMEC-1 cells, either uninfected or infected with *R. rickettsii*, were incubated in culture medium at 34°C as described elsewhere [21,30]. Briefly, cells were lysed in 1 x PBS buffer (containing 0.1% Triton X-100, protease and phosphatase inhibitors) for 15 min on ice [36]. Lysates were centrifuged at 6,000 x *g* for 5 min to separate the rickettsial secreted effectors and host cytosolic proteins (cytoplasmic fraction) from the intact rickettsiae and insoluble host proteins (pellet fraction). The cytoplasmic fraction was filtered through a 0.45-μm pore size filter (Millipore), and pellet fraction was resuspended into 1 x PBS (containing protease and phosphatase inhibitors). Samples from the pellet (P) and cytoplasmic (C) fractions were immunoblotted with anti-RLip, anti-Pat1 (positive control for secreted *R. rickettsii* effector), anti-OmpA/B (as control for *R. rickettsii* surface protein), or anti-GAPDH Abs (host cytoplasmic control protein) [21,30].

### Expression and purification of recombinant RLip proteins

The **R. rickettsii* RLip* (gene locus_tag: A1G_01170) gene was codon-optimized (CO) for *E. coli* expression and cloned into the bacterial expression vector pET30a with N-terminal 6x-His epitope tag by GeneScript. Mutation of the catalytic active site at position 138 (Ser 138 to Ala 138) of RLip was introduced via the QuikChange II XL site-directed mutagenesis kit (Agilent technologies, Cat-No.: 200521−5) according to the manufacturer’s instructions using the Forward primer 5’-CTTTTAACACCAGCGGCCACGCGCTGGGCGCGATTATC-3’ and Reverse primer 5’-GATAATCGCGCCCAGCGCGTGGCCGCTGGTGTTAAAAG-3’. The constructed mutant plasmid pET30a-RLip-S138A-CO was confirmed by sequencing. Both pET30a-RLip-WT-CO and RLip-S138A-CO plasmids were transformed into *E. coli* BL21-Codon Plus expression host and grown at 37°C for overnight in LB media supplemented with Kanamycin (30 µg/ml). Protein expression was induced using 0.5 mM IPTG for 16 hrs at 20°C. Proteins were purified using HisPur^TM^ Ni-NTA magnetic beads according to manufacturer’s instructions and identity of the recombinant proteins was confirmed by western blot analysis using an anti-His Ab (Fig 2A) as well as by MS analysis (CVID core facility, University of Maryland School of Medicine, Baltimore, MD, USA).

**Fig 1:**
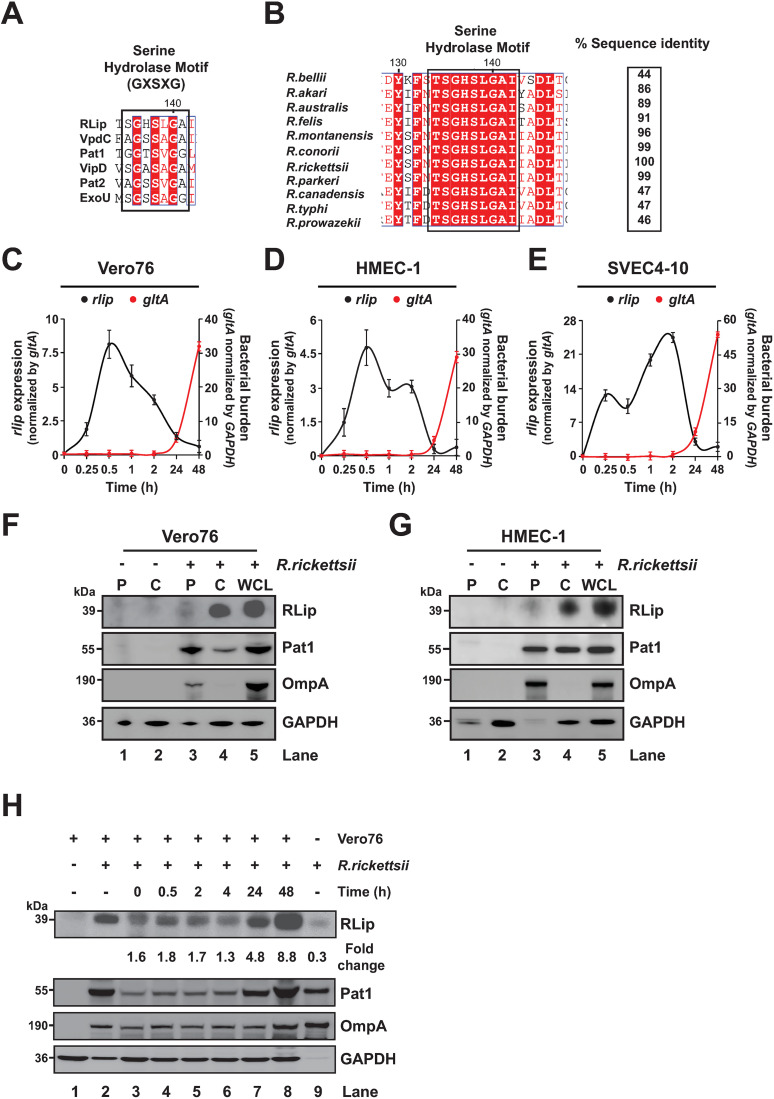
RLip is a secreted effector harboring a conserved hydrolase motif. (A) Comparative sequence alignment of catalytic Serine hydrolase motifs among rickettsial proteins [Pat1, Pat2, and *Rickettsia* Lipase (RLip, locus_tag: A1G_01170)], and other bacterial lipases VipD (*Legionella pneumophila*), VpdC (*Legionella pneumophila*), and ExoU (*Pseudomonas aeruginosa*). Highly conserved amino acids of the Serine hydrolase motif are highlighted in red. (B) Sequence alignment of the Serine hydrolase motif (GXSXG) and the percent sequence identity of RLip protein (*R. rickettsii*) with RLip molecules from other *Rickettsia* species are shown. (C–E) Expression kinetics of *rlip* (black line) and bacterial burden (red line) during *R. rickettsii-*infection of Vero76 (C), HMEC-1 (D), and SVEC 4-10 (E) was determined by RT-qPCR. *RLip* expression was normalized with respect to *gltA* transcription level. Bacterial burden was determined by normalized *gltA* transcription level with respect to host cell *GAPDH* transcription level as described previously [31,38]. Uninfected or *R. rickettsii*-infected Vero76 (F) or HMEC-1 (G) cells were lysed with 0.1% Triton X-100 treatment and separated into cytoplasmic (C) and pellet (P) fractions. Samples were immunoblotted with anti-RLip, anti-Pat1 (secreted rickettsial effector control), anti-OmpA/B (rickettsiae associated surface protein control), or anti-GAPDH Abs (host cytoplasmic protein control). Whole cell lysates (WCL) were used as expression control for all target proteins. (H) Partially purified rickettsiae were incubated with Vero76 cells for various length of time at 34°C. The harvested whole host lysates were analyzed by immunoblotting as described in the Materials and Methods section. Uninfected, *R. rickettsii-*infected WCL as well as partially purified rickettsiae were used as controls. Densitometry in panel H was performed using Fiji software, and RLip as well as Pat1 expression was normalized with respect to levels observed in partially purified *Rickettsia* (lane 9). Data shown in panel H is presented as fold change ratios between RLip/Pat1. Error bars (C–E) represent means ± standard error of the mean (SEM) from 3 independent experiments. Images in panels F-I are a representative of 3 independent experiments.

### Lipase activity assay

The lipase activity of rRLip-WT-CO and rRLip-S138A-CO proteins was measured using a calorimetric lipase assay kit as per manufacturer’s instruction (Abcam, Cat-No.: ab102524). Briefly, the lipase assay measures the breakdown of triglyceride substrate into free fatty acids and glycerol, which is quantified enzymatically via monitoring a linked change in the OxiRed probe absorbance (OD_570 nm_). Freshly purified rRLip-WT-CO and rRLip-S138A-CO proteins were buffer exchanged with the lipase assay buffer provided with the kit. For host cell lysate preparation, monolayers of HMEC-1 cells were harvested and washed with 1 x PBS. Host cell pellets were resuspended in 0.5 ml of assay buffer, lysed by sonication and supernatants were collected by centrifugation at 10,000 x *g* for 15 min at 4°C. The protein concentration of supernatants was determined by BCA protein assay kit. For lipase assay, 25 µg of rRLip-WT-CO or rRLip-S138A-CO protein was mixed with 50 µg of HMEC-1 cell lysate and incubated with the reaction buffer containing lipase substrate, OxiRed probe, and enzyme mixture. Reactions were measured at an OD_570 nm_ for 90 min, at intervals of 2 min, at 37°C. The Lipase activity of the rRLip-WT-CO and rRLip-S138A-CO proteins was calculated following manufacturer’s instruction.

### Lipase membrane assay

Lipid membranes were obtained from Echelon (Cat-No.: P-6001) and assay was performed according to the manufacturer’s instructions. Briefly, 1 μg of 6x-His-tagged rRLip-WT-CO, rRLip-S138A-CO or rLacZ (non-specific binding control) protein was spotted onto the membrane and incubated for 1 h at room temperature. Membranes were washed using 1 x PBS-Tween 20 (0.1%), and membranes were probed with the anti-His Ab for 1 h at room temperature, followed by another incubation period of 1 h with HRP-conjugated Ab.

### Yeast cytotoxicity assay

The *R. rickettsii* genome encoded wild-type *RLip* gene was cloned by PCR into the *SacI* and *XhoI* sites of yeast expression vector pYES2/CT with C-terminal epitope (V5 and 6x-His) tags (S4 Fig) according to manufacturer’s instruction, using Forward primer 5’-TTAAGCTTGGTACCGAGCTCATGCCTACGTA CAAAAATTCTAAACATATTAGCAC-3' and Reverse primer 5’-GCCCTCTAGACTCGAGTTAACATATTA

GAGGATATAGATGATAATTACTTATATCTCCTGTTGT-3’. Constructed plasmid pYES2/CT-RLip-WT_GS_ [carrying RLip encoded by *R. rickettsii* wild-type (WT) genome sequence (GS)] was confirmed by sequencing. Next, the codon-optimized (CO) **R. rickettsii* RLip* gene (optimized for *E. coli* expression; GeneScript) was subcloned by PCR into the *SacI* and *XhoI* sites of yeast expression vector pYES2/CT (S4 Fig) using Forward primer 5’-TTAAGCTTGGTACCGAGCTCATGCCCACATATAAAAATTCAAAGC

ACATATCC-3’ and Reverse primer 5’-GCCCTCTAGACTCGAGGTGGTGATGATGGTGGTGACAAATC

AGCGG-3’ and constructed plasmid pYES2/CT-RLip-WT-CO was confirmed by sequencing. In addition, RLip-S138A-CO mutant was subcloned into the *SacI* and *XhoI* sites of yeast expression vector pYES2/CT (S4 Fig) and constructed plasmid pYES2/CT-RLip-S138A-CO was confirmed by sequencing as described above.

The constructed plasmids were transformed in *S. cerevisiae* strain INVSc-1 using Frozen-EZ Yeast Transformation II^TM^ Kit (Zymo Research, Cat-No.: T2001), following the manufacturer’s instructions. The transformed yeast cells were grown in synthetic complete (SC) medium agar without Uracil and containing 2% glucose (SC-U + Glu) to select plasmids at 30°C for 3 days. Plasmid containing yeast cells were grown in SC-U + Glu media overnight at 30°C. The culture was pelleted, washed, and resuspended in SC-U without a carbon source. The resuspended yeast cells were induced and/or repressed in agar medium containing 2% galactose (SC-U + Gal) and/or 2% glucose (SC-U + Glu) respectively and incubated for 3 days at 30°C. For CFU assay, the resuspended yeast transformants were serially diluted in SC-U without a carbon source and plated on inducing (SC-U + Gal) and repressing (SC-U + Glu) agar. After incubation at 30°C for 3 days, the colonies were counted to determine the percentage of CFU on inducing agar with respect to that on repressing agar.

### Plasmid generation and transposon mutagenesis of RLip

We generated an enhanced construct for Himar1-based transposon mutagenesis of *R. parkeri* (pEGTn02) based on pMW1650 [37] that moves the *E. coli* origin of replication out of the transposed sequence and adds the following elements. The GFP gene was replaced with the gene encoding the brighter fluorescent protein AausFP1, the rifampicin resistance gene with replaced with the spectinomycin resistance gene, and ssrA protein degradation tags were incorporated next to the inverted terminal repeats (ITRs). Fragments of the transposed sequence were synthesized (IDT) and inserted into the pJTM1650 plasmid (gift of Rebecca Lamason, Department of Biology, Massachusetts Institute of Technology, Cambridge, MA, USA) by standard digestion and ligation methods using *E. coli* as a cloning host. The sequence of pEGTn02 was verified by whole plasmid sequencing. Prior to electroporation into *R. parkeri*, we created a barcoded pEGTn02 library, adding unique 20-nt barcodes to simplify tracking of mutants in downstream applications. To do this, pEGTn02 was linearized by *PspOMI* digestion and gel purified. The purified vector (0.005 pmol/µL) was combined with a single-stranded DNA barcode oligo (0.2 pmol/µL; 5’-GCGAGCTCTCACTAGAGGATCCACATANNNNNNNNNNNNNNNNNNNNAGCCGCCATGGGTCGCTACTAGTTCCG-3’) in a NEBuilder HiFi DNA Assembly reaction. The assembly reaction was incubated at 50°C for 15 min and transformed into chemically competent *E. coli* DH5α cells. Transformants were selected on LB agar supplemented with kanamycin. Plasmid DNA was isolated from individual colonies and sequenced to confirm successful barcoding. Each clone contained a unique 20-nt barcode sequence between the *PspOMI* restriction sites. The complete sequence and map of the construct is shown in S7 Fig.

Transposon mutagenesis was conducted by electroporating freshly prepared electrocompetent *R. parkeri* strain (Portsmouth) with ~1 µg of barcoded pEGTn02. After electroporation, the bacteria were used to infect a 6-well plate seeded with a confluent monolayer of Vero76 cells. Infection was carried out for 30 min with rocking at 37°C. After infection, an overlay of DMEM with 2% (v/v) FBS and 0.5% (w/v) ultra-pure agarose was added to each well. The culture was then incubated overnight at 34°C, and 5% CO_2_. The next day, a 1 mL overlay of agarose was added with 50 µg/mL of spectinomycin. The culture was returned to incubate at 34°C, 5% CO_2_ until plaques formed. Plaques were picked and frozen in 200 µL of Brain Heart Infusion broth at −80°C until processed. For expansion, 100 µL of the resuspended plaque was used to infect one well of a 6-well plaque with confluent Vero76 cells. After the infection reached completion, the bacteria were harvested. A sample was set aside for storage at −80°C, and another was used for PCR mapping of the transposon insertion site.

To map the transposon insertion site, semi-random PCR was conducted on the boiled lysate of the purified and expanded *R. parkeri* transposon mutant. This process utilized specific primers for the transposon sequence EGExTn1 (5’-AACAATTAAATACTCCTTTAGGTGGTGGTC-3’) and EGExTn2 (5’-TCGTTGATCAAAGCTCGCCGCG-3’) and a universal primer (Univ1, 5’-GGCCACGCGTCGACTAGTACNNNNNNNNNNGATAT-3’). The Univ1 primer consisted of a specific, G/C-rich 20-nt, 5’ anchor, a random 10-nt sequence, and a 3’ pentamer. After the first round of PCR, the amplified DNA was extracted using the Monarch Spin PCR and DNA Cleanup Kit. Next, nested-PCR was conducted using primers for the transposon EGInTn1 (5’-TGGCGCCGGCGCAAGCACATACTATATTATCTAAAGATCC-3’) and EGInTn2 (5’-ATATCACTGTGTGGCTTCAGGCCGCCATCCACTGCG-3’) and the 5’-anchor (Univ2, 5’-GGCCACGCGTCGACTAGTAC-3’). The amplified DNA was extracted again and sent for Sanger sequencing. Insertions sites were determined by using BLAST against the *R. parkeri* Portsmouth genome to identify the genomic sequence adjacent to the transposon. Our findings showed that the transposon insertion disrupts the *rlip* coding sequence at nucleotide position 694 of 1062, creating a truncated RLip protein of about 28 kDa (Fig 4B).

**Fig 2:**
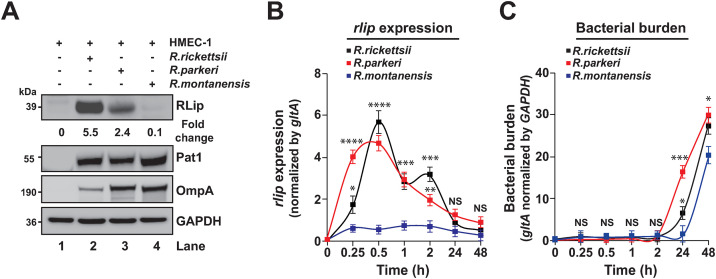
RLip facilitates intracellular replication in HMEC-1 cells. (A–C) HMEC-1 cells were infected with spotted fever group (SFG) rickettsiae, including *R. rickettsii*, *R. parkeri*, and *R. montanensis* (non-pathogenic) for up to 48 hrs. (A) Host cell lysates were analyzed by western blot analysis using anti-RLip, anti-Pat1, anti-OmpA/B, or anti-GAPDH Abs. Expression kinetics of *rlip* (B) and bacterial burden (C) during infection of *R. rickettsii*, *R. parkeri*, and *R. montanensis* was determined by RT-qPCR. *Rlip* expression was normalized with respect to *gltA* transcription level. Bacterial burden was determined by normalized *gltA* transcription level with respect to host cell *GAPDH* transcription level. Densitometry in panel A was performed using Fiji software, while RLip and Pat1 expression was normalized with respect to levels in uninfected HMEC-1 cell lysates (lane 1) and were presented as fold change ratios between RLip/Pat1. Error bars (B, C) represent SEM from 3 independent experiments, NS, not significant; *P ≤ 0.05, **P ≤ 0.01, ***P ≤ 0.005, ****P ≤ 0.001. Images in panels A are a representative of 3 independent experiments.

**Fig 3:**
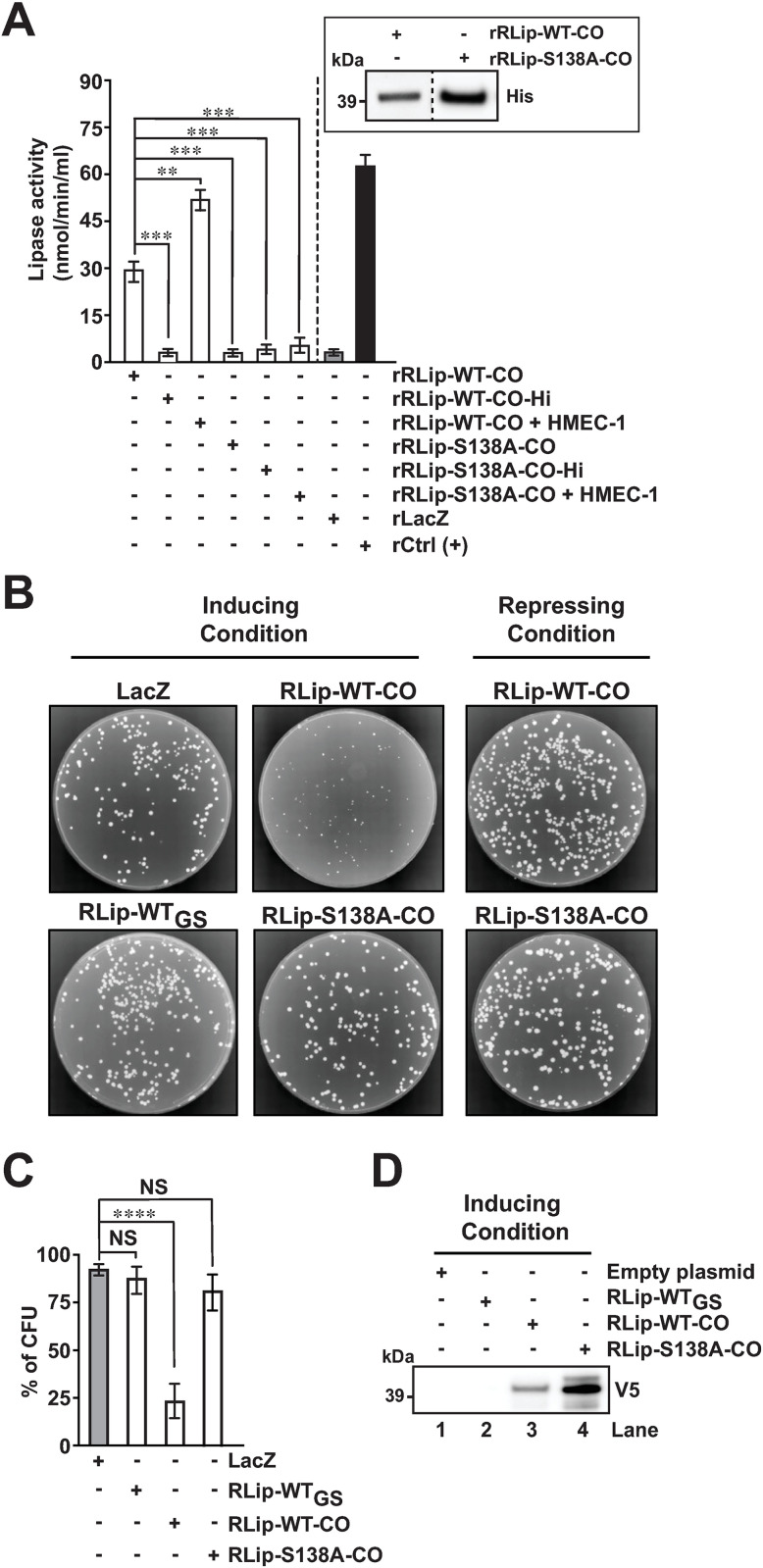
RLip is a rickettsial effector with lipase and cytotoxic activities. (A) Lipase activity of purified codon-optimized recombinant (r) rRLip-WT-CO, rRLip-S138A-CO and heat-inactivated rRLip-WT-CO-Hi, rRLip-S138A-CO-Hi proteins in the absence or presence of HMEC-1 host cell lysate was assessed as described previously [20,21]. The rLacZ protein and a lipase derived from *Chromobacterium* were used as a non-specific protein and positive (+) control, respectively. Inset represents a western blot analysis of the utilized rRLip-WT-CO and rRLip-S138A-CO using an anti-His Ab. (B) Transformed yeast cells were streaked onto inducing (SC-U + Gal) or repressing (SC-U + Glu) agar and incubated at 30°C for 3 days. (C) Cytotoxicity assay in yeast strain INVSc1 transformed with plasmids expression RLip-WT_GS_, RLip-WT-CO or lipase mutant RLip-S138A-CO was performed as described previously [20,21]. Colonies were counted to determine the percentage of colony formation units (CFU) on inducing agar with respect to that on repressing agar. LacZ plasmid was used as control in panels B, and C. (D) Western blot analysis of V5-epitop-tagged RLip-WT_GS_, RLip-WT-CO or lipase mutant RLip-S138A-CO expression in yeast strain INVSc1 under inducing conditions (SC-U + Gal medium). The total proteins from yeast cells carrying the appropriate plasmid were probed with anti-V5 Ab. Error bars in panels A, and C represent means ± SEMs (standard errors of the means) from 3 independent experiments; NS, not significant; **P ≤ 0.01, ***P ≤ 0.005, ****P ≤ 0.001. Images in A, B, and D are a representative of 3 independent experiments.

### Immunofluorescent assay (IFA)

Eight-well chamber slides were seeded with HMEC-1 cells (~ 50 x 10^4^ cells per well) and infected with partially purified *R. rickettsii*, *R. parkeri* WT or *R. parkeri rlip*::Tn mutant strain (MOI = 20 [2 hrs] or 5 [24 hrs] as described previously [28,30,31,38]. Briefly, rickettsiae were added to HMEC-1 cells and incubated for various length of time at 34°C with 5% CO_2_. Following incubation, cells were washed with 1 x PBS and fixed with 4% PFA for 20 min at room temperature. Cells were then permeabilized in blocking buffer (0.3% saponin and 0.5% normal goat serum in 1 x PBS) for 30 min and incubated for 1 h with the following primary Abs diluted in antibody-dilution buffer (0.3% saponin in 1 x PBS): anti-*Rickettsia* (1:100, guinea pig serum), and anti-LAMP2 (1:100). Cells were then washed with 1 x PBS and incubated for 1 h with Alexa Fluor conjugated Abs (1:1,500) in Ab-dilution buffer. Next, cells were washed with 1 x PBS and mounted with ProLong Gold antifade mounting medium containing DAPI.

For RLip neutralization experiments, we distinguished extracellular from intracellular bacteria by differential staining as described previously [30]. Briefly, antibody-treated *R. rickettsii*-infected cells were stained first with anti-*Rickettsia* Ab in 1 x PBS containing 0.5% normal goat serum for 1 h at room temperature and then incubated with Alexa Fluor 488 in 1 x PBS containing 0.5% normal goat serum for 1 h. Next, cells were permeabilized in blocking buffer (0.3% saponin and 0.5% normal goat serum in 1 x PBS) and stained with anti-*Rickettsia* Ab for 1 h, followed, by incubation with an Alexa Fluor 594 Ab for 1 h, and mounted using ProLong Gold antifade medium containing DAPI.

Images were acquired using the Nikon W-1 spinning disk confocal microscope (University of Maryland School of Medicine, Confocal Core Facility, Baltimore, MD, USA) and analyzed using Fiji software as described previously [30,31,38]. The percentage of internalized bacteria (approximately 200 bacteria were counted per strain and time point) was calculated by dividing the number of extracellular bacteria by the total number of bacteria, multiplying by 100, and then subtracting this number from 100% to get the percentage of intracellular bacteria.

### Cellular viability assay

Host cellular cytotoxicity was assessed by the lactate dehydrogenase (LDH) assay as previously described [38] Briefly, supernatants (50 µl) from untransfected HeLa cells or cells transfected with pcDNA4-Flag empty vector, pcDNA4-Flag-RLip-WT, or pcDNA4-Flag-RLip-S138A were collected at 24 hrs post-transfection and then mixed with 50 µl of LDH Detection buffer (LDH-Glo™ Cytotoxicity Assay, Promega). Reactions were incubated for 1 h at room temperature and luminescent signal was measured using the Varioskan LUX microplate reader (Thermo Fisher Scientific). Precent cytotoxicity was calculated following manufacturer’s instruction.

### Antibody-mediated neutralization of RLip

Partially purified *R. rickettsii* was pre-treated with 50 µg of affinity purified anti-RLip, or pre-immune IgG for 30 min on ice. Pretreated rickettsiae were added onto HMEC-1 monolayer and incubated for various length of time at 34°C and 5% CO_2_. Rickettsial invasion was monitored by IFA as well as real-time reverse transcription-quantitative PCR (RT-qPCR).

### RNA isolation and quantitative real-time PCR

Bacterial burden [assessed by rickettsial housekeeping citrate synthase (*gltA*) gene expression, normalized with respect to host housekeeping gene *GAPDH* transcriptional levels] and *rlip* gene expression was determined after *R. rickettsii*, *R. parkeri* WT or *R. parkeri rlip*::Tn mutant infection in various cell lines by RT-qPCR [20,31,38]. In this effort, host cells infected with either *R. rickettsii, R. parkeri* WT or *R. parkeri rlip*::Tn mutant were collected at 0.25, 0.5, 1, 2, 24, and 48 hrs post-infection. RNA was isolated from ~ 1 x 10^6^ cells using Quick-RNA^TM^ miniprep kit (Zymo research, Cat-No.: ZR1055). cDNA was synthesized from the RNA samples using GoScript™ Reverse Transcription System (Promega) as per manufacturer’s instructions. After cDNA synthesis, amplification reaction was carried out by iQ^TM^ SYBR^®^ Green Supermix (Bio-Rad), using primer pair Prlip-1 (Fwd) (5’-CATTCACTTGGAGCAATAATTGCAG-3’) and Prlip-2 (Rev) (5’-AGTTGTTACTTTTCCGGTAAATAA-3’) for rickettsial *rlip*. Expression of host cell housekeeping gene, *GAPDH*, and rickettsial *gltA*, was determined as described previously [31,38]. Cycling conditions were as follows: 1 cycle at 95°C for 3 min; 40 cycles at 95°C for 15 sec, 55°C for 15 sec, and 72°C for 20 sec; and 1 cycle to generate the dissociation curve. The RT-qPCR amplification and detection were performed on an QuantStudio™ 3 Real-Time PCR Systems (Applied Biosystem by Thermo Fischer). The specificities of these primer pairs were verified by PCR on DNA isolated from *R. rickettsii* and *R. parkeri* WT.

*RLip* expression was normalized with respect to *gltA* transcriptional levels, while bacterial burden (number) was determined by *gltA* expression, normalized with respect to *GAPDH* transcriptional levels, following the equation: Normalized expression *= E* ^*-ΔCT*^, where *E* = efficiency of PCR and ΔC_T_ = C_T_ *target* − C_T_ *reference* as described previously [31,38].

### Evaluation of the *rlip* gene in *R. parkeri* WT and *R. parkeri rlip*::Tn

To test for *rlip* gene expression in *R. parkeri* WT and *R. parkeri rlip*::Tn strains we isolated genomic (g)DNA from Vero76 cells infected with *R. parkeri* WT and *R. parkeri rlip*::Tn mutant strain (48 hpi) using the Trizol method. PCR reactions using gDNA from WT and *rlip*::Tn mutant samples were carried out using primers Prlip-3 (Fwd) (5’-TGATGATAAACGTACGATGCCTACGTACAAAAATTCTAAACATAT TAGCAC-3’) and Prlip-4 (Rev) (5’-TACCTCATCAGCGCGCACATATTAGAGGATATAGATGATAATTA CTTATATCT-3’) to amplify RLip, while Pat1 was detected using primer pair Pat1 (Fwd) (5’-CCTTTCAGATATAATACAAGG-3’) and Pat1 (Rev) (5’-GTCATTAGCATATACTCCACCATC-3’) and GltA was amplified using GltA (Fwd) (5’-CCTTTCAGATATAATACAAGG-3’) and GltA (Rev) (5’-ATGATTTATGGGGAACTACC-3’). Samples were analyzed by agarose gel electrophoresis.

### Extract preparation and western blot analysis

*Rickettsia*-infected Vero76, HMEC-1, or SVEC4–10 cells were lysed for 2 hrs at 4°C in ice-cold lysis buffer (50 mM HEPES [pH 7.4], 137 mM NaCl, 10% glycerol, 1 mM EDTA, 0.5% NP-40, and supplemented with protease and phosphatase inhibitor cocktails) as described previously [30]. Equal amounts of protein were loaded onto an SDS-PAGE and membranes were probed with anti-RLip, anti-Pat1, anti-V5, anti-His, anti-GAPDH, and anti-OmpA/B Abs, followed by enhanced chemiluminescence with HRP-conjugated secondary Abs.

### Statistical analysis

The statistical significance was assessed using analysis of variance with Bonferroni’s procedure and Student’s *t*-test. Data are presented as mean ± standard error of the mean (SEM), unless stated otherwise and asterisks denote statistical significance as: *P ≤ 0.05; **P ≤ 0.01; ***P ≤ 0.005; ****P ≤ 0.001, compared with the indicated controls. Statistical analyses were performed using GraphPad PRISM, version 9.

## Results

### RLip is a secreted effector with a putative lipase domain

As an obligate intracellular parasite, *Rickettsia* requires membranolytic enzymes to escape membrane-bound vacuoles, to gain access to the metabolite-rich host cytosol. Recent reports from others and our laboratory have demonstrated the importance of rickettsial membranolytic effectors in facilitating host cell colonization [20,21,23,24,39,40]. However, the sporadic presence or dispensability of some of these rickettsial effectors [22–25] may indicate the presence of additional membranolytic enzyme(s) involved in the invasion process of host cells. To test the hypothesis that rickettsiae possess additional membranolytic effector(s), we searched the rickettsial genomes using the web-based Phyre2 program [26] and identified in the *R. rickettsii* (Sheila Smith) genome, with ~99% confidence, a putative secreted lipase (locus_tag: A1G_01170) with a Serine hydrolase motif (GXSXG), which we named RLip (*Rickettsia* Lipase). The predicted putative lipase, RLip (of 353 amino acids), possesses a conserved Serine hydrolase motif (GXSXG) [41,42], as depicted by the sequence alignment of RLip with other lipases, including ExoU (*Pseudomonas aeruginosa*) [13]; VipD (*Legionella pneumophila*) [15], VpdC (*Legionella pneumophila*) [16], and rickettsial Pat1 as well as Pat2 [20,21] (Fig 1A). Further sequence comparison shows that the Serine hydrolase motif was conserved amongst RLip molecules of spotted fever group (SFG), transitional group, ancestral group, and typhus group rickettsiae (Fig 1B).

As lipases belong to the α/β hydrolase family with an active site Serine situated at the catalytic loop between α-helices and a β-sheet [43–45], we generated a homology model of RLip using the Phyre2 software [46]. Our model depicted that RLip harbors a classical lipase structure consisting of a β-sheet surrounded by two α-helices (S1A Fig) with a catalytic Serine placed in the catalytic loop (S1A Fig; inset). Further comparison of the lipase structure with other phospholipases (ExoU, VipD, VpdC, Pat1, as well as Pat2) provided additional evidence that RLip harbors a conserved lipase motif (S1B Fig). Of note, all presented models were validated by Ramachandran plot using the WinCoot 0.9.8.95 EL software [34] (S2 Fig).

Given that phospholipases have been implicated to play an important role during host cell infection [20,21,23,24,39,40], we sought to determine the transcriptional pattern of RLip during *R. rickettsii-*infection of various mammalian cells by RT-qPCR. We used Vero76 cells as one of the most commonly utilized epithelial cell line to propagate and study some aspects of rickettsiae biology. Endothelial cells of human (HMEC-1) and mouse origin (SVEC 4–10) were used to study intracellular pathogenesis as they represent primary target cells for rickettsial infection [1,22,47]. Our data demonstrated that RLip transcription (displayed as *rlip* expression, normalized by *gltA* levels) is rapidly increased during the early stages of invasion [0.25–2 hrs post-infection (hpi)] within all three tested host cells and remained expressed during the course of infection [20,32] (Fig 1C–E). Bacterial burden (as depicted by *gltA* expression, normalized by *GAPDH* levels) appeared to increase after the doubling time (8–10 hrs) of rickettsiae at 24 hpi (Fig 1C–E). These findings indicate that RLip likely plays a critical role during the rickettsial host infection process. To define the function of RLip during host infection, we raised an antibody against the RLip protein (anti-RLip) and determined its specificity using uninfected and *R. rickettsii*-infected Vero76, and HMEC-1 cells as well as purified recombinant (r)RLip protein (S3 Fig). Our data showed that the rRLip protein (S3A Fig, lanes 3–5), expressed in *E. coli*, has a slightly faster mobility as compared to that from *R. rickettsii*-infected host cell lysates (S3A or S3B Fig, lane 2), indicating that differences in post-translational modification and/or sample composition from two different sources might account for the observed mobility shift changes of RLip. Next, we examined if RLip is secreted from *R. rickettsii* during infection of Vero76 (Fig 1F) and HMEC-1 (Fig 1G) cells by performing cellular fractionation into cytoplasmic and pellet fractions. The cytoplasmic [(C), carrying host soluble and rickettsial secreted proteins] and pellet [(P), carrying rickettsiae and insoluble host debris] fractions were analyzed by immunoblotting as described previously [30]. We observed that glyceraldehyde-3-phosphate dehydrogenase (GAPDH; host cytoplasmic protein) appeared in the cytoplasm of both uninfected and infected cells (Fig 1F and 1G, lanes 2 and 4, respectively). Of note, the observed faint GAPDH bands within both pellet fractions are likely the result of incomplete lysis of the host cells or residual supernatants left with the pellet fractions (Fig 1F and 1G, lanes 1 and 3, respectively). OmpA, a rickettsial outer membrane protein [30,48], was only detected in the pellet fraction of infected cells (Fig 1F and 1G, lane 3), suggesting that lysis of host cells in the presence of 0.1% Triton X-100 is not affecting the cell surface integrity of *R. rickettsii*, which is in agreement with previous findings [30]. Furthermore, Pat1 was present in both the pellet (Fig 1F and 1G, lane 3) and cytoplasm (Fig 1F and 1G, lane 4) of infected cells, implying that Pat1 is partially secreted into the host cell cytoplasm. RLip was secreted into the cytoplasm of infected Vero76 or HMEC-1 cells, while minimally retained by the bacteria itself (Fig 1F and 1G, lane 4 vs. 3, respectively). Due to its intriguing expression and secretion pattern, we tested the hypothesis whether RLip expression was induced upon host cell invasion. In this effort, we infected Vero76 cells for various length of time with partially purified *R. rickettsii* from host cells and analyzed RLip protein expression by western blot analysis. Our data revealed that RLip was rapidly induced as soon as purified rickettsiae were added to the host cells (0 hpi) (Fig 1H, lane 9 vs. 3). Furthermore, RLip remained expressed during the early stages of infection [up to 4 hpi (Fig 1H, lanes 4–6)], reaching maximum levels after the doubling time (8–10 hrs) of rickettsiae at 24–48 hpi (Fig 1H, lanes 7–8). Of note, RLip expression profile was evaluated with respect to Pat1 levels as OmpA expression did not represent the observed bacteria replication kinetics (Fig 1H).

#### RLip may contribute to the intracellular replication of rickettsiae in HMEC-1 cells.

Next, we sought to evaluate the protein expression profile of RLip during host cell infection among spotted fever group (SFG) rickettsiae, which included two pathogenic (*R. rickettsii* and *R. parkeri*), and the non-pathogenic *R. montanensis* strain. Intriguingly, although all three species encode a putative full-length RLip lipase (of 353 amino acids), with either 99% or 96% identity when compared to RLip protein of *R. rickettsii* (Fig 1B), our findings showed that RLip was predominantly expressed during *R. rickettsii* or *R. parkeri* infection, while its expression was minimally detected during *R. montanensis* infection (Fig 2A). To test if the observed difference in RLip expression may account for variations of bacterial burden, we evaluated the *rlip* expression and replication kinetics by RT-qPCR. Our data revealed comparable expression kinetics of *rlip* for *R. rickettsii* and *R. parkeri* in infected host cells, with *rlip* level being the highest during the early stages (0.25–2 hpi), while its expression remained low and unchanged for *R. montanensis* infection (Fig 2B). In addition, we found that all three *Rickettsia* species grow within HMEC-1 cells, although the levels of bacterial burden in *R. rickettsii*-, and *R. parkeri*-infected cells were higher (24–48 hrs) as compared to that from *R. montanensis-*infected cells (Fig 2C). In sum, these data show that RLip is differentially expressed among pathogenic and non-pathogenic rickettsiae and may contribute to the infection process of pathogenic *Rickettsia* species.

### RLip possess lipase and cytotoxic activities

Our bio-informatic analysis predicted RLip as a putative lipase (Fig 1). To further interrogate its functional characteristics, we generated codon optimized full-length wild-type His-tagged recombinant RLip protein (rRLip-WT-CO) as well as a catalytic active site mutant, rRLip-S138A-CO, in which a Serine (S) was mutated to an Alanine (A) at position 138 (Fig 3A). As previously demonstrated for Pat1 and Pat2 [20,21,24], our data revealed that rRLip-WT-CO possesses lipase enzymatic activity, while mutagenesis of the S138 residue significantly reduced the activity (Fig 3A). Of note, heat-inactivation (Hi) of either rRLip-WT-CO or rRLip-S138A-CO resulted in the loss of the lipase activity (Fig 3A). To test if host cell factor(s) contribute to the lipase activity of RLip, we measured the enzymatic activities of rRLip-WT-CO or rRLip-S138A-CO in the presence of uninfected HMEC-1 cell lysate and found that the addition of host cell lysate enhanced the activity of rRLip-WT-CO but not that of rRLip-S138A-CO (Fig 3A). These findings suggest that RLip possesses lipase activity, which is enhanced by host cell factor(s).

During invasion, intracellular bacteria employ effector molecules to target host (eukaryotic) cellular processes to establish a replication niche. However, the identification and biological characterization of these effectors has been difficult, often limited by the lack of genetically tractable systems. Heterologous model systems, such as *Saccharomyces cerevisiae*, provide an effective tool to identify, and assess biological functions of bacterial effector proteins and their putative roles in pathogenesis [49]. In fact, work from others and our laboratory have used *S. cerevisiae* as a genetically tractable system to demonstrate the cytotoxic effects of various effectors, including among others ExoU (*P. aeruginosa*) [13,50], Pat1, and Pat2 (*Rickettsia*) [20,21]. To assess the cytotoxicity of RLip, we cloned RLip-WT-CO and RLip-S138A-CO into the pYES2/CT vector (S4 Fig) and performed the yeast cytotoxicity assay as previously described [20,21]. Of note, pYES2/RLip-WT_GS_ [carrying *RLip* encoded by *R. rickettsii* WT genome sequence (GS)] and pYES2/CT/LacZ plasmids were used as controls (S4 Fig). Yeast transformants carrying either pYES2/CT/LacZ or pYES2/RLip-WT_GS_ grew well under either inducing (SC-U + Gal) or repressing conditions (SC-U + Glu) (Fig 3B and 3C). However, yeast carrying pYES2/RLip-WT-CO showed a significant reduction in growth under inducing condition (SC-U + Gal), while yeast carrying pYES2/RLip-S138A-CO showed no growth inhibition (Fig 3B and 3C). Further evaluation of RLip-WT-CO and RLip-S138A-CO protein expression within the yeast transformants by western blot analysis confirmed that both proteins were expressed (Fig 3D). However, the yeast transformants carrying the pYES2/RLip-WT_GS_ plasmid [harboring the wild-type AT-rich GS of *RLip*] showed no protein expression (Fig 3D), suggesting that the rickettsial AT-rich coding sequence of RLip is not expressed in yeast heterologous system [21]. Next, we sought to evaluate the cytotoxic activity of RLip in mammalian host by transfecting HeLa cells with empty vector, wild-type RLip (pcDNA4-Flag-RLip-WT) or active site mutant RLip (pcDNA4-Flag-RLip-S138A) and assess the levels of cellular cytotoxicity using a LDH assay as described previously [38]. Our data revealed that RLip-WT expression significantly enhanced the level of cell death as compared to cells expressing the RLip-S138A protein (S5 Fig). Collectively, our data suggest that RLip possesses both lipase and cytotoxic activities, which require a functional Serine hydrolase motif.

### RLip recognizes various phosphoinositides

During host invasion, intracellular bacteria target phosphoinositides (PIs), a family of signaling lipids that play important roles in membrane dynamics and regulating intracellular trafficking [22,30,51]. In fact, intracellular pathogens employ numerous effectors to modulate the PI metabolism in order to evade microbicidal host defense responses and to establish a successful host colonization [6,22,30,52–54]. Recent reports from others and our laboratory, demonstrated that rickettsial effectors, like Pat1, Pat2, and RISK-1 target host lipids metabolism and promote the escape from membrane-bound vacuoles into host cytosol for replication [20,21,24,30]. Given these prior reports as well as our current data showing that RLip possesses lipase activity (Fig 3), we tested the binding selectivity of rRLip-WT-CO or rRLip-S138A-CO towards phosphoinositides (PIs) using a protein-lipid panel as described previously [30]. RLip bound preferentially to PIs {phosphatidylinositol 3-phosphate [PI(3)P], phosphatidylinositol 4-phosphate [PI(4)P], phosphatidylinositol 5-phosphate [PI(5)P], phosphatidylinositol 3,4-bisphosphate [PI(3,4)P_2_], phosphatidylinositol 3,5-bisphosphate [PI(3,5)P_2_], phosphatidylinositol 3,4,5-trisphosphate [PI(3,4,5)P_3_], phosphatidylinositol 4,5-bisphosphate [PI(4,5)P_2_]} (S6 Fig). In contrast, RLip did not recognize phosphatidylinositol [PI], phosphatidylethanolamine (PE), phosphatidylcholine (PC), phosphatic acid (PA), phosphatidylserine [PS], Lysophosphatidic acid (LPA), Lysophosphocholine (LPC), and sphingosine 1-phosphate (S1P) (S6 Fig). These data indicate that RLip can directly engage specific PIs without requiring a functional Serine hydrolase motif.

### RLip facilitates intracellular survival of rickettsiae by promoting the evasion from lysosomal destruction

**Fig 4:**
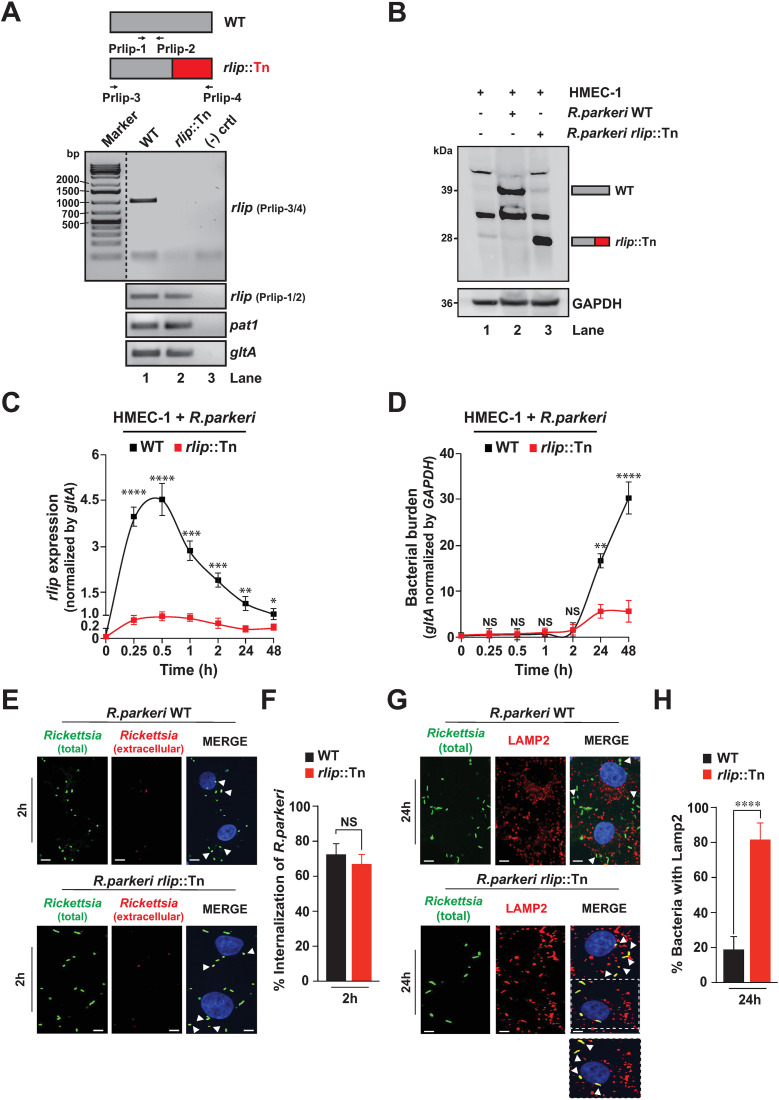
RLip contributes to *R. parkeri* intercellular survival by facilitating the escape from phagosomes. (A) Evaluation of the intact *rlip* gene and *rlip* transcript using *R. parkeri* WT or *R. parkeri rlip*::Tn bacterial DNA. (B) RLip protein expression was detected in HMEC-1 cells infected with *R. parkeri* WT or *R. parkeri rlip*::Tn bacteria by western blot analysis using anti-RLip, and anti-GAPDH Abs. (C, D) *R. parkeri* WT- or *R. parkeri rlip*::Tn mutant-infected HMEC-1 cells were analyzed for *rlip* expression (C) and bacterial burden (D) by RT-qPCR. The gene expression of RLip was normalized with respect to *gltA* transcription level, while bacterial burden (*gltA*) was normalized with respect to host cell *GAPDH* transcription level. (E, F) HMEC-1 cells were infected with purified *R. parkeri* WT and *R. parkeri rlip*::Tn mutant (MOI: 20) and internalization was assessed at 2 hpi by IFA. (G-H) Colocalization of *R. parkeri* WT or *R. parkeri *rlip**::Tn mutant (MOI: 5) with LAMP2 was evaluated by IFA at 24 hpi. Nuclei were stained with 4’,6-diamidino-2-phenylindole (DAPI). Numbers of extracellular and intracellular rickettsiae (E, F) as well as colocalization between rickettsiae and LAMP2 (G-H) was analyzed using Coloc 2 plugin Fiji software. Bars in panels E, and G, 10 μm. Approximately 200 bacteria-infected cells were counted per condition and time point. Error bars (C, D, F and H) represent means ± standard error of the mean (SEM) from three independent experiments; NS, not significant; *P ≤ 0.05, **P ≤ 0.01, ***P ≤ 0.005, ****P ≤ 0.001. Images in A and B are a representative of 3 independent experiments.

To test the biological role of RLip during rickettsial host invasion, we would like to highlight that using traditional genetic manipulation approaches to study gene-specific functions are not readily amenable for obligate intracellular bacteria and only current efforts in creating transposon mutant libraries in rickettsiae have resulted in the generation of some validated gene-deficient mutants in *R. parkeri* [29]. Accordingly, we isolated a Himar1 transposon insertion mutant of *R. parkeri* that mapped to the *rlip* gene (MC1_RS01085) [*rlip*::Tn]. The transposon insertion disrupts the *rlip* coding sequence, which we expect would disrupt the function of RLip (Fig 4A). We evaluated the presence of the intact *rlip* gene by PCR using *R. parkeri* WT or *R. parkeri rlip*::Tn bacterial DNA and the primer pair Prlip-3/4 (Fig 4A). Consistent with the transposon mapping, our data revealed that the intact *rlip* gene was detected in *R. parkeri* WT but not in *R. parkeri rlip*::Tn bacteria, while *pat1* and *gltA* were present in both *R. parkeri* WT and *R. parkeri rlip*::Tn bacteria (Fig 4A). Next, we determined the protein expression of RLip in HMEC-1 cells infected with *R. parkeri* WT or *R. parkeri rlip*::Tn bacteria using western blot analysis (Fig 4B) and showed that *R. parkeri* WT-infected cells expressed the full-length RLip protein of 39 kDa, while HMEC-1 cells infected with *R. parkeri rlip*::Tn bacteria displayed a truncated RLip protein of about 28 kDa (Fig 4B). We further monitored the transcriptional pattern of RLip (displayed as *rlip* expression) and bacterial burden (using rickettsial *gltA* expression) during infection of HMEC-1 cells using *R. parkeri* WT and *R. parkeri rlip*::Tn bacteria by RT-qPCR. Using a primer pair (Prlip-1/2) that was designed to detect a transcript of RLip in both *R. parkeri* WT and *R. parkeri rlip*::Tn bacteria (Fig 4A), we showed that in cells infected with *R. parkeri* WT, RLip transcription increased during the early stages of invasion (0.25–2 hpi) and remained expressed during the course of infection (Fig 4C). However, no appreciable changes in RLip transcript level were observed in the cells infected with the *R. parkeri rlip*::Tn mutant strain (Fig 4C). Bacterial burden in HMEC-1 cells infected with *R. parkeri* WT appeared to increase after the doubling time of rickettsiae at 24 hpi (Fig 4D), following a similar kinetics observed in cells infected with *R. rickettsii* (Figs 1D and 2C). In contrast, rickettsial replication was significantly impaired in host cells that were infected with *R. parkeri rlip*::Tn (Fig 4D) suggesting that RLip plays a critical role during the rickettsial host infection process.

We further evaluated whether the observed difference in bacterial replication was due to variances in internalization by assessing the percentage of host cells infected with purified *R. parkeri* WT and *R. parkeri rlip*::Tn bacteria using differential staining to distinguish between extracellular (tethered) and intracellular bacteria as described previously [30]. Our data revealed comparable levels of internalization for both *R. parkeri* WT and *R. parkeri rlip*::Tn bacteria in HMEC-1 cells (Fig 4E and 4F). As the observed difference in rickettsial growth may indicate a defect in vacuolar escape, we next evaluated the importance of RLip during phagosomal escape by immunofluorescence staining using the lysosomal marker, LAMP2 [30]. We infected HMEC-1 cells with purified *R. parkeri* WT or *R. parkeri rlip*::Tn bacteria and assessed the colocalization of rickettsiae with LAMP2 and found that, unlike *R. parkeri* WT, *R. parkeri rlip*::Tn bacteria colocalized with LAMP2, suggesting that *RLip*-deficient *Rickettsia* remain enclosed within phagosomes (Fig 4G and 4H). As a complementary approach, we performed an antibody-mediated neutralization assay to assess the percentage of host cells infected by partially purified *R. rickettsii* pre-treated with anti-RLip Ab, with respect to cells treated with pre-immune IgG, as described previously [30]. Our IFA showed comparable levels of rickettsial internalization in host cells pre-treated with anti-RLip Ab or pre-immune IgG (S8A and S8B Fig). As rickettsiae minimally retained RLip protein outside of host cells and rapidly induce it as soon as the bacteria encounters host cells (Fig 1F and 1H), we further assessed the role of RLip during HMEC-1 infection by employing an anti-RLip Ab pretreatment. Our data revealed a significant decrease in bacterial load at 24 hpi for *R. rickettsii* pre-treated with the anti-RLip Ab as compared to that with pre-immune IgG (S8C Fig), indicating that anti-RLip Ab-treatment of *R. rickettsii*, negatively affected rickettsial growth in host cells. Furthermore, we evaluated the phagosomal escape of rickettsiae by IFA using LAMP2 staining and observed that anti-RLip Ab-treated *R. rickettsii* mostly colocalized with the LAMP2 marker, suggesting the rickettsiae remain enclosed within phagosomes (S8D and S8E Fig). In contrast, *R. rickettsii* treated with pre-immune IgG did not colocalize with LAMP2 implying the successful escape from lysosomal destruction (S8D and S8E Fig). Taken collectively, this report shows, via two independent approaches; one using *R. parkeri rlip*::Tn mutant and another with anti-RLip antibody pretreatment of the bacteria, that RLip facilitates rickettsial intracellular survival by contributing to the escape from phagolysosomal destruction into host cytoplasm.

## Discussion

*Rickettsia* species overcome host defense responses to establish a successful intracytosolic replication niche. In fact, evasion of phagolysosomal destruction is essential for rickettsial survival and is mediated by their membranolytic effectors [20,21,23,24,39,40]. However, some of the rickettsial membranolytic enzymes have been reported to be dispensable depending on the host cell type, while others are sporadically present in rickettsial genome [22–25,55], suggesting the presence of alternative enzymes required for disrupting the vacuolar membrane during host invasion. In this study, our bioinformatic analysis using the web-based Phyre2 program [26] identified a putative secreted lipase with a Serine hydrolase motif (GXSXG) in the *R. rickettsii* (Sheila Smith) genome, which we named RLip. The sequence analyses showed that the Serine hydrolase motif was conserved among RLip molecules identified in *Rickettsia* species. The RLip expression analyses revealed that the protein was expressed during pathogenic rickettsiae infection, however, its expression was minimally detected during *R. montanensis* infection, respectively, suggesting a selective role of RLip in facilitating the intracellular colonization of pathogenic *Rickettsia* species into host cells. Furthermore, the data revealed that RLip expression after infection with *R. rickettsii* increases rapidly upon contact with host cells and remained expressed during infection, suggesting that RLip likely acts to facilitate bacterial intracellular trafficking and replication.

The secretion of rickettsial effectors involve various types of secretion pathways, which include the Sec translocon dependent type V secretion system (T5SS), type I secretion system (T1SS), type IV secretion system (T4SS) and others [22,55–57]. Intriguingly, analysis of the RLip protein sequence by the web-based SignalP-6.0 program [35] did not predict a N-terminal signal sequence required for protein secretion via Sec translocon. In addition, the RLip protein was not captured by a RvhD4 coimmunoprecipitation assay performed in our prior reporting [30], suggesting that the molecule may not be an effector of the T4SS. However, using cellular fractionation assays, we demonstrated that RLip was secreted into the host cytoplasm during infection and being minimally retained by the *Rickettsia*. These findings would indicate that either the T1SS or any other unappreciated protein secretion pathways could be involved in the secretion process of RLip. To our knowledge these data also provide the first description of a rickettsial effector that is synthesized during infection, secreted into host cytoplasm and retained minimally by the bacteria. Intriguingly, RLip expression and secretion patterns shows similarities to the chlamydial protease- or proteasome-like activity factor (CPAF) [58] and hypothetical protein CT795 [59]. In fact, CPAF, like RLip, is secreted during infection and remains hardly associated with purified *Chlamydia* and therefore was designated as an infection-dependent antigen [58,59]. Thus, we hypothesis that RLip may acts as a secreted rickettsial infection-dependent antigen, which will be addressed in our future work.

Bacterial membranolytic effectors have been shown to play a critical role in modulating membrane dynamics during infection. In this study, we are reporting that RLip effector has an active site Serine at position 138, required for its lipase enzyme activity and cytotoxicity. Moreover, we found that the lipase activity of RLip was increased in the presence of host cell lysates suggesting the involvement of a host factor(s) to promote its biological activity during host invasion. These findings corroborate with earlier reports showing that eukaryotic factor(s) enhanced the enzymatic activities of both rickettsial lipases, Pat1 and Pat2 [20,21,24]. However, the identities of those eukaryotic co-factor(s) are unknown, which warrants further investigation. Furthermore, the lower lipase enzymatic activity of RLip observed in our assay, might be necessary to support the obligate intracellular lifestyle of rickettsiae, without inflicting any rapid damage to host cells, at least before rickettsiae exit infected cells to promote further transmission to neighboring cells/organs of the host.

It is also important to highlight that the biological relevance of rickettsial membranolytic effectors in helping the infection process seems to depend on the host cell-type and the specific *Rickettsia* species [22–25,55]. For instance, Pat2 is sporadically present in rickettsial genomes, while Pat1 is highly conserved in all *Rickettsia* species [22,55]. Intriguingly, Pat1 shows to be redundant for rickettsial growth in HMEC-1 cells but required for survival in bone-marrow derived macrophages (BMDM) [24]. To test our hypothesis that *Rickettsia* requires additional enzyme(s) to escape from vacuolar membrane, our bioinformatic search identified a new membranolytic enzyme, RLip, which has a conserved Serine hydrolase motif in all *Rickettsia* species. We further assessed the role of RLip by employing infection assays using *R. parkeri* WT or *R. parkeri* [*rlip*::Tn] bacteria and observed that the *rlip* transcript levels increased in cells infected with *R. parkeri* WT during the early stages of invasion (0.25–2 hpi), while cells infected with the *R. parkeri rlip*::Tn mutant strain showed no upregulation during the course of infection. Furthermore, we observed that *R. parkeri* WT-infected cells expressed the full-length RLip protein, while HMEC-1 cells infected with *R. parkeri rlip*::Tn mutant bacteria displayed a truncated RLip protein with comparable abundance (Fig 4B). The observed lack of correlation between mRNA and protein abundance could be explained by the possibility that the Himar1 transposon insertion caused a destabilization effect on the *rlip* transcript leading to its rapid degradation. Intriguingly, we recently observed such phenomenon of a lesser stable transcripts relative to protein lifespan for the rickettsial effector, Pat1, during host cell infection [21]. Moreover, it is not uncommon to observe a lack of correlation between mRNA and protein abundance of certain genes for some prokaryotes, which is linked to a longer half-life of a given protein relative to mRNA [60,61].

Another intriguing observation we made was that the bacterial burden in HMEC-1 cells infected with *R. parkeri* WT appeared to increase after the doubling time of rickettsiae at 24 hpi (Fig 4D), while the replication level was significantly decreased in cells infected with *R. parkeri rlip*::Tn (Fig 4D) suggesting that RLip plays a critical role during the rickettsial host infection process. In fact, our colocalization analysis, using the lysosomal marker, LAMP2, showed that the observed decrease in bacterial burden is likely a direct result of lysosomal-dependent destruction of the *R. parkeri rlip*::Tn mutant due to its inability to timely escape phagosomes prior to their fusion with lysosomes. Intriguingly, findings from other laboratories suggested that Pat1 is dispensable for rickettsial growth in HMEC-1 cells [24], indicating a compensatory involvement of another lipase. Thus, it is tempting to speculate that RLip possibly compensates for the loss of Pat1 to ensure bacterial growth in HMEC-1 cells, while both lipases are likely required to facilitate successful rickettsial survival and replication in immune defense cells, like macrophages [24]. However, the precise mechanism of how Pat1 and RLip contribute to the intracellular life cycle of *Rickettsia* requires further investigation.

To further validate the role of RLip during host infection, we performed an anti-RLip Ab-mediated neutralization assay during *R. rickettsii*-mediated infection of HMEC-1 cells and showed that disruption of RLip function impairs bacterial intracellular survival. These data provide additional cooperating evidence to our infection data using *R. parkeri rlip*::Tn mutant bacteria and collectively support a role for RLip in the escape from lysosomal destruction. Intriguingly, preceding findings from our laboratory showed that pre-treatment of *R. typhi* with either anti-Pat1 or anti-Pat2 Ab affected rickettsial infection as well as delayed rickettsial phagosome escape [21]. These data suggest that rickettsial entry into the host does not require RLip function, however, likely involves other effectors, like hemolysins and lipases (TlyC, Pat1, Pat2, or Pld) [20–24,62,63], and additional molecules, including Sca’s [30,64–68] and RalF [28,69].

**Fig 5:**
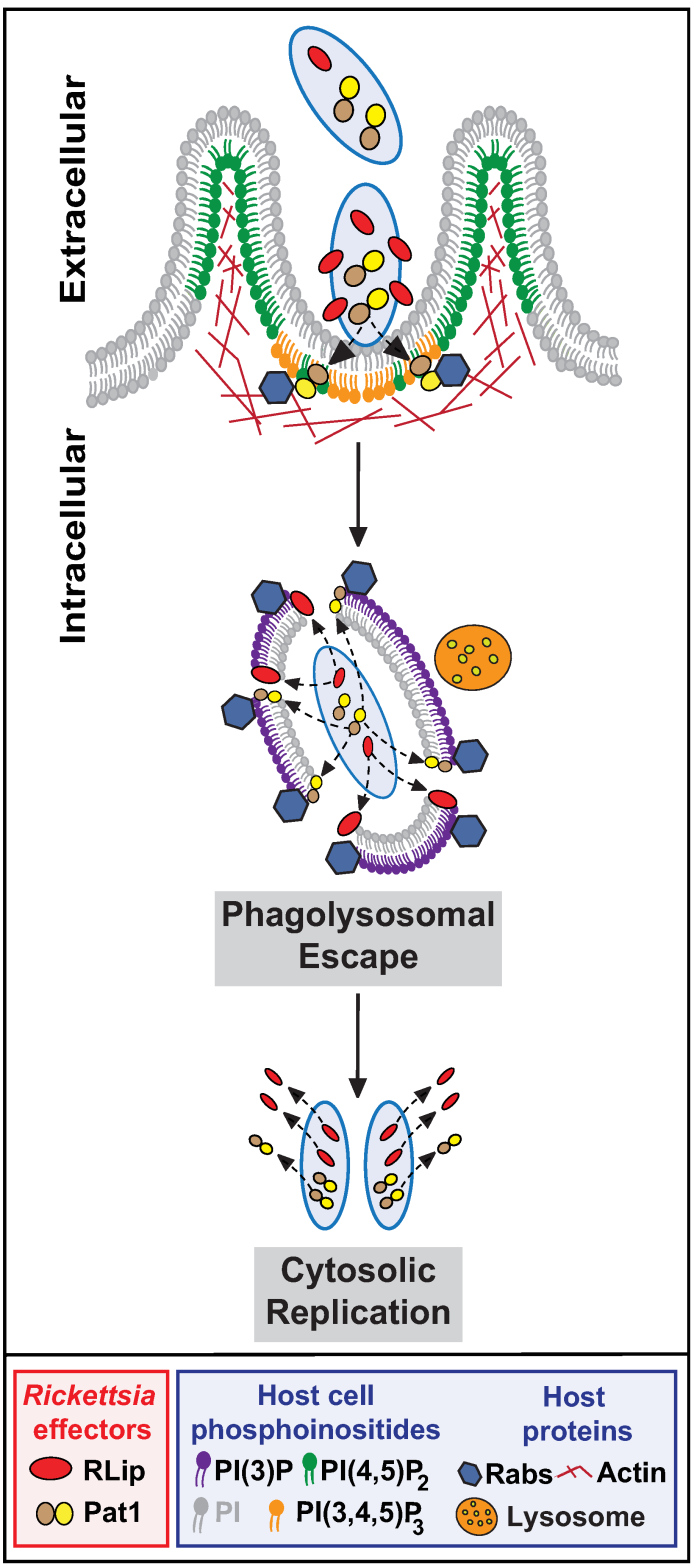
Working model for RLip facilitating pathogenic rickettsiae intracellular survival. Expression of RLip is low during the cell-free stage, while rapidly induced as soon as the bacteria encounters the host cell. Upon host cell invasion, RLip is predominately released into the host cytoplasm, while minimally retained by the bacteria itself. Our findings suggest that RLip plays an important role in the phagosomal escape of pathogenic *Rickettsia* species into the host cell cytoplasm to facilitate the establishment of a replication niche.

Finally, and as iterated above, the rickettsial vacuolar escape from lysosomal fusion into host cytosol is a process involving many effector molecules: Pld [23,39], Pat1 [20,21,24], Pat2 [20,21], TlyA [39], TlyC [39,63], RISK-1 [30] and RalF [28,30,69]. However, it is important to note that the biological activity of these membranolytic effectors: ***i)*** differs based on the host cell type, ***ii)*** varies depending on the stage of rickettsial intracellular lifestyle, or ***iii)*** might have functional redundancy [22–25,55]. Collectively, the data presented in this manuscript suggest that RLip alone or in conjunction with other rickettsial effector(s) facilitates bacterial escape from vacuolar membranes (Fig 5).

## Supporting information

S1 Fig RLip harbors a putative lipase structure. (A) Homology model of RLip, showing a classical lipase structure of a β-sheet surrounded by two α-helices, was constructed using the Phyre2 software [46]. (B) Homology models of lipase structures from other bacterial phospholipases (ExoU, VipD, VpdC, and rickettsial Pat1, or Pat2) were constructed as described above.
(TIF)

S2 Fig Validation of the modeled RLip lipase structure using WinCoot. Ramachandran plot analysis was performed using the WinCoot 0.9.8.95 EL software [34] and homology models of RLip (*R. rickettsii*), and other bacterial lipases, including Pat1 (*R. typhi*), Pat2 (*R. typhi*), VipD (*L. pneumophila*), VpdC (*L. pneumophila*), and ExoU (*P. aeruginosa*) were displayed with PyMOL. All residues, except glycine (Gly) and proline (Pro) are plotted as circles, Gly are shown as triangles, and Pro as squares. Residues in allowed and preferred regions are colored in blue, outliers are shown in red. The Ramachandran plot shows the favored region in salmon color, allowed region in beige color, and disallowed region (outlier) in grey color. Percentage of amino acid (AA) residues that resided within the favored, allowed, or outlier region are shown below each plot.
(TIF)

S3 Fig Specificity validation of the generated anti-RLip antibody. The recombinant RLip-WT protein, encoded by codon-optimized *RLip* gene (locus_tag: A1G_01170) was used to generate an anti-RLip Ab (RLip). The specificity of the anti-RLip Ab was validated by western blot analysis using whole cell lysates (WCL) of uninfected (lane 1) and *R. rickettsii*-infected (lane 2) Vero76 (A) or HMEC-1 cells (B). Purified recombinant (r)RLip-WT protein expressed in *E. coli* (A; lanes 3–5; 10–500 ng) was included as control. Immunoblotting with anti-OmpA/B and anti-GAPDH Abs was used to control for uninfected and *R. rickettsii*-infected cell lysates (A, and B; lanes 1 and 2). Images are a representative of 3 independent experiments.
(TIF)

S4 Fig Vector maps used for yeast transformation. The empty yeast expression vector pYES2/CT with C-terminal epitope (V5 and 6x-His) tags (A), plasmid containing *R. rickettsii* genome sequence (GS) of the wild-type (WT) RLip gene (pYES2/CT-RLip-WT_GS_, B), and plasmids containing either codon-optimized (CO) gene encoding RLip-WT (pYES2/CT-RLip-WT-CO, C) or RLip-S138A-CO (pYES2/CT-RLip-S138A-CO, D) were used for transformation into *S. cerevisiae* strain INVSc-1, to perform the cytotoxicity assay.
(TIF)

S5 Fig RLip exhibits cytotoxicity in mammalian cells. (A, B) Cellular cytotoxicity was evaluated in untransfected or HeLa cells transfected with pcDNA4-Flag empty vector, pcDNA4-Flag-RLip-WT, or pcDNA4-Flag-RLip-S138A. (A) Cell pellets were collected 24 hrs post-transfection, lysed, and analyzed for RLip-WT and RLip-S138A expression by immunoblotting using anti-Flag and anti-GAPDH Abs. (B) Supernatants from the same experiment were utilized to measure the cellular cytotoxicity by using lactate dehydrogenase (LDH) release assay following manufacturer’s instructions. Cytotoxicity levels from RLip-WT or RLip-S138A transfected cells were normalized by values from untransfected and empty vector transfected cells. Images shown in panel A is a representative of 3 independent experiments. Error bars in panel B represent means ± SEMs (standard errors of the means) from 4 independent experiments; **P ≤ 0.01.
(TIF)

S6 Fig RLip binds specific phosphoinositides. Lipid membrane assays (Echelon) were performed as per manufacturer’s instructions. The membrane was spotted with 1 μg of purified 6x-His-tagged rRLip-WT or rRLip-S138A protein and incubated for 1 h at room temperature. 6x-His-tagged rLacZ protein was used as a non-binding control. Binding of RLip to phosphoinositides was detected using an anti-His and HRP-conjugated Ab. The lipid membrane assay is a representative of 3 independent experiments.
(TIF)

S7 Fig Vector map and sequence information of the generated RLip transposon mutant. (A) Vector map of barcoded pEGTn02 plasmid used to generate the RLip transposon mutant. (B) Selected sequence information is highlighted by color: ITR, SsrA, PrpsL, specR, PompA, AausFP1, Barcode.
(TIF)

S8 Fig Antibody-mediated neutralization of RLip affects survival and phagosomal escape of *R. rickettsii.* Partially purified *R. rickettsii* were pre-treated with 50 µg of affinity purified anti-RLip, or pre-immune IgG for 30 min on ice. Pretreated rickettsiae were added onto HMEC-1 monolayer and incubated for various length of time at 34°C and 5% CO_2_. (A, B) Extracellular and intracellular rickettsiae were assessed at 2 hpi by IFA using a MOI: 20 via differential staining using Alexa Fluor-488- and −594-conjugated anti-*Rickettsia* guinea pig serum as described in the Materials and Methods section. (C) Bacterial burden of antibody-treated rickettsiae in infected HMEC-1 cells was assessed for various length of time by rickettsial housekeeping citrate synthase (*gltA*) gene expression using RT-qPCR. *GltA* expression was normalized with respect to *GAPDH* transcription level as described in the Materials and Methods section. (D-E) Colocalization of antibody-treated *R. rickettsii* with LAMP2 was evaluated by IFA at 24 hpi using a MOI: 5 and Alexa Fluor-488-conjugated anti-LAMP2 Ab and Alexa Fluor-594-conjugated anti-*Rickettsia* guinea pig serum. Inset shows a close-up representation of *Rickettsia*-LAMP2 staining of HMEC-1 cells infected with anti-RLip Ab treated bacteria at 24 hpi. The cell nuclei were stained with 4’,6-diamidino-2-phenylindole (DAPI). Numbers of extracellular and intracellular rickettsiae (A-B) as well as colocalization between *Rickettsia* and LAMP2 (D-E) was analyzed using Coloc 2 plugin Fiji software. Bars in panels A, and D, 10 μm. Approximately 200 bacteria-infected cells were counted per condition and time point. Error bars (B, C, and E) represent means ± standard error of the mean (SEM) from 3 independent experiments; NS, not significant; **P ≤ 0.01.
(TIF)

S9 Fig Unmodified Images for Main Figures. Fig 1F, G: Uninfected or *R. rickettsii*-infected Vero76 (F) or HMEC-1 (G) cells were separated into cytoplasmic (C) and pellet (P) fractions. Samples were immunoblotted with anti-RLip, anti-Pat1, anti-OmpA/B, or anti-GAPDH Abs. Whole cell lysates (WCL) were used as expression control for all target proteins. Fig 1H: Partially purified rickettsiae were incubated with Vero76 cells for various length of time and whole host lysates were analyzed by immunoblotting as described in the Materials and Methods section. Fig 2A: HMEC-1 cells were infected with spotted fever group (SFG) rickettsiae, including *R. rickettsii*, *R. parkeri*, and *R. montanensis* for up to 48 hrs and analyzed by western blot analysis using anti-RLip, anti-Pat1, anti-OmpA/B, or anti-GAPDH Abs. Fig 4A, B: (A) Evaluation of the intact *rlip* gene and *rlip* transcript using *R. parkeri* WT or *R. parkeri rlip*::Tn bacterial DNA. (B) RLip protein expression was detected in HMEC-1 cells infected with *R. parkeri* WT or *R. parkeri rlip*::Tn bacteria by western blot analysis using anti-RLip, and anti-GAPDH Abs. Red highlighted area presents the cropped images shown in the main figures.
(PDF)

S10 Fig Unmodified Images for Supporting Figures. S3A, B Fig: The recombinant RLip-WT protein was used to generate an anti-RLip Ab (RLip). The specificity of the anti-RLip Ab was validated by western blot analysis using whole cell lysates (WCL) of uninfected (lane 1) and *R. rickettsii*-infected (lane 2) Vero76 (A) or HMEC-1 cells (B). Purified recombinant (r)RLip-WT protein expressed in *E. coli* (A; lanes 3–5; 10–500 ng) was included as control. S5A Fig: Cellular cytotoxicity was evaluated in untransfected or HeLa cells transfected with pcDNA4-Flag empty vector, pcDNA4-Flag-RLip-WT, or pcDNA4-Flag-RLip-S138A. (A) Cell pellets were collected 24 hrs post-transfection, lysed, and analyzed for RLip-WT and RLip-S138A expression by immunoblotting using anti-Flag and anti-GAPDH Abs. Red highlighted area presents the cropped images shown in the supporting figures.
(PDF)
