# Supplementary figures and images for: Pathogenic rickettsiae encode a secreted lipase that facilitates intracytosolic colonization in host cells
Source: PLoS One. 2025 Oct 8;20(10):e0332810. doi: 10.1371/journal.pone.0332810 (PMC12507273; doi:10.1371/journal.pone.0332810)

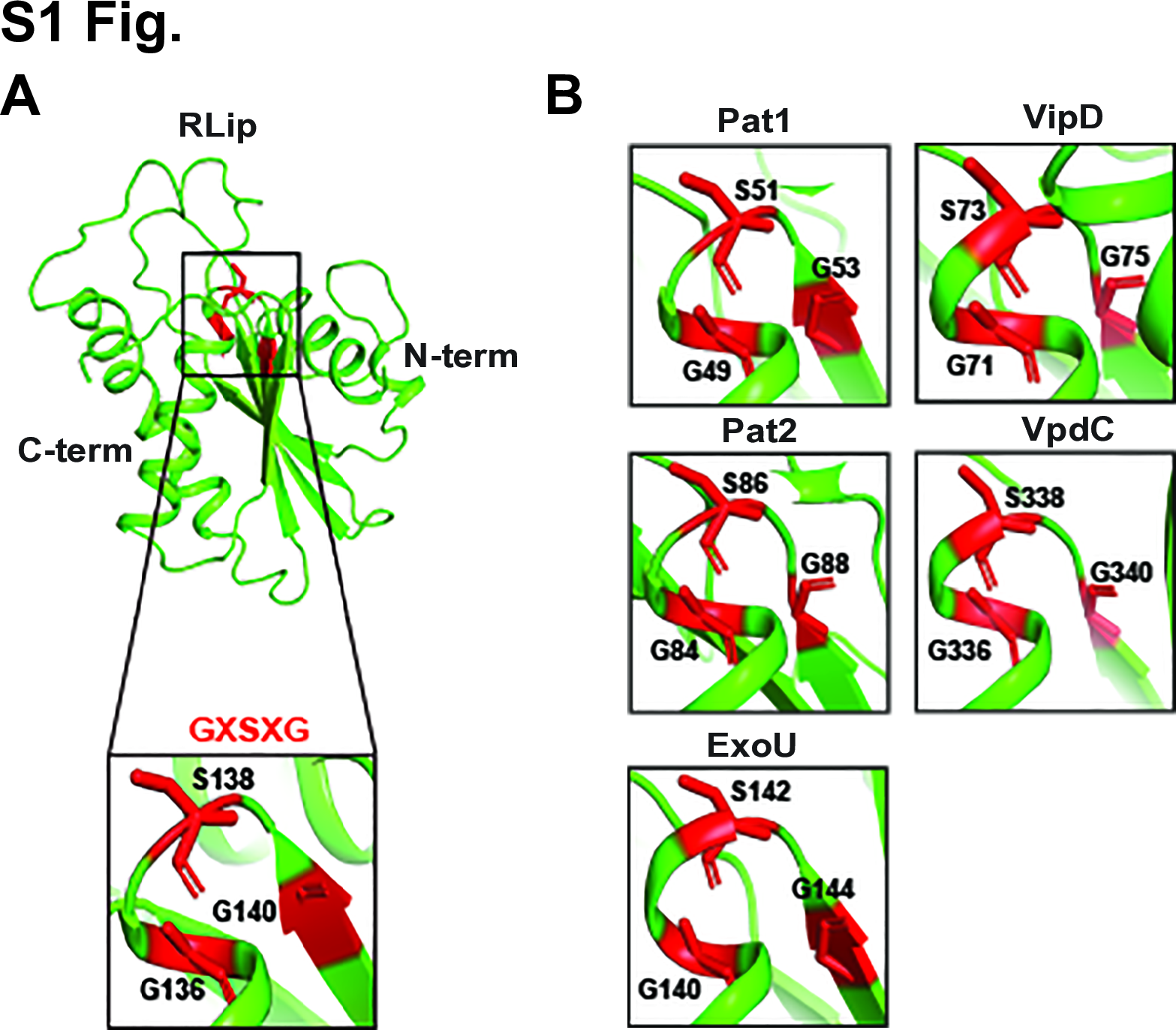

Supplement: S1 Fig — (A) Homology model of RLip, showing a classical lipase structure of a β-sheet surrounded by two α-helices, was constructed using the Phyre2 software [46]. (B) Homology models of lipase structures from other bacterial phospholipases (ExoU, VipD, VpdC, and rickettsial Pat1, or Pat2) were constructed as described above. (TIF) [file pone.0332810.s001.tif]

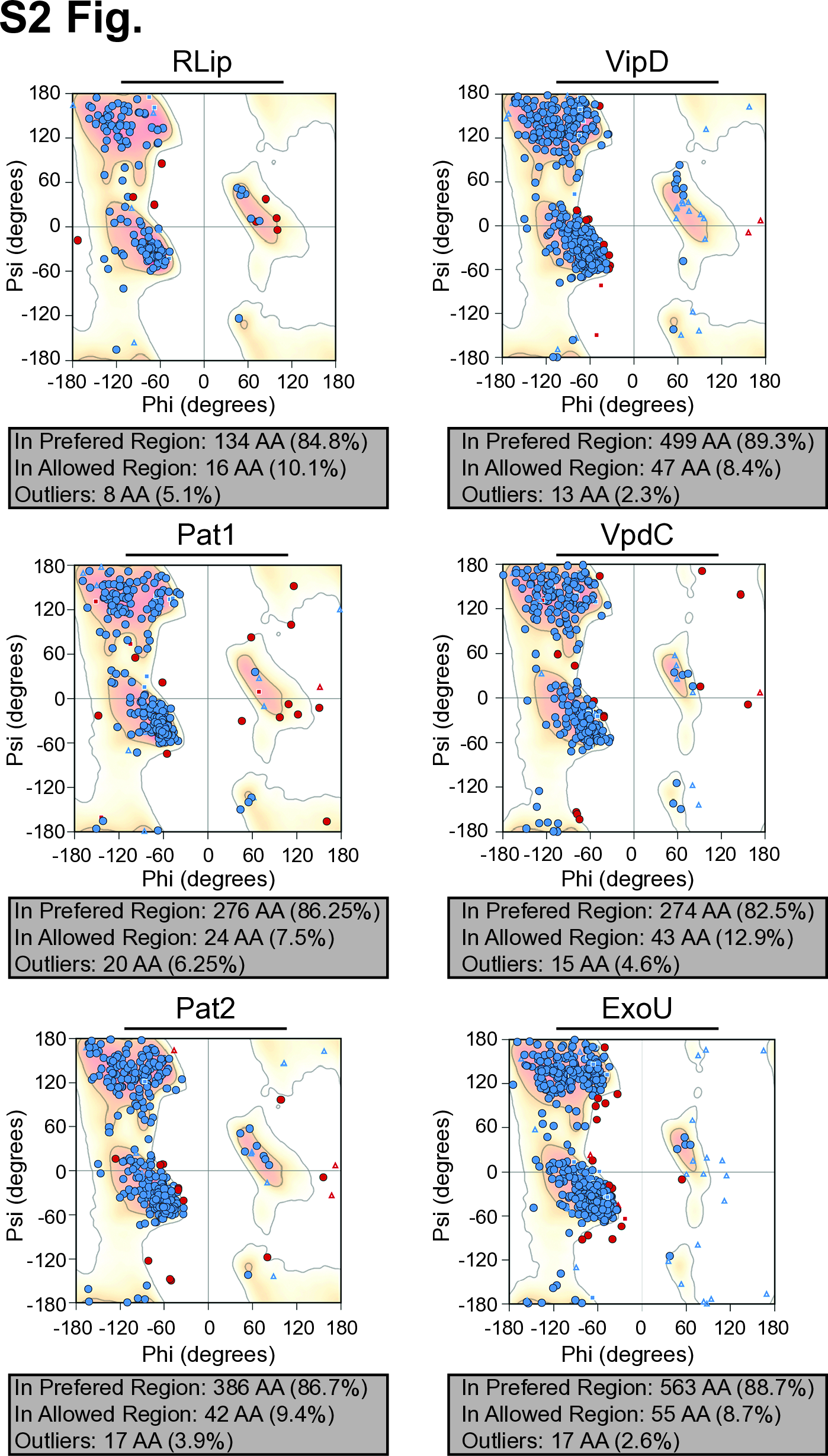

Supplement: S2 Fig — Ramachandran plot analysis was performed using the WinCoot 0.9.8.95 EL software [34] and homology models of RLip (R. rickettsii), and other bacterial lipases, including Pat1 (R. typhi), Pat2 (R. typhi), VipD (L. pneumophila), VpdC (L. pneumophila), and ExoU (P. aeruginosa) were displayed with PyMOL. All residues, except glycine (Gly) and proline (Pro) are plotted as circles, Gly are shown as triangles, and Pro as squares. Residues in allowed and preferred regions are colored in blue, outliers are shown in red. The Ramachandran plot shows the favored region in salmon color, allowed region in beige color, and disallowed region (outlier) in grey color. Percentage of amino acid (AA) residues that resided within the favored, allowed, or outlier region are shown below each plot. (TIF) [file pone.0332810.s002.tif]

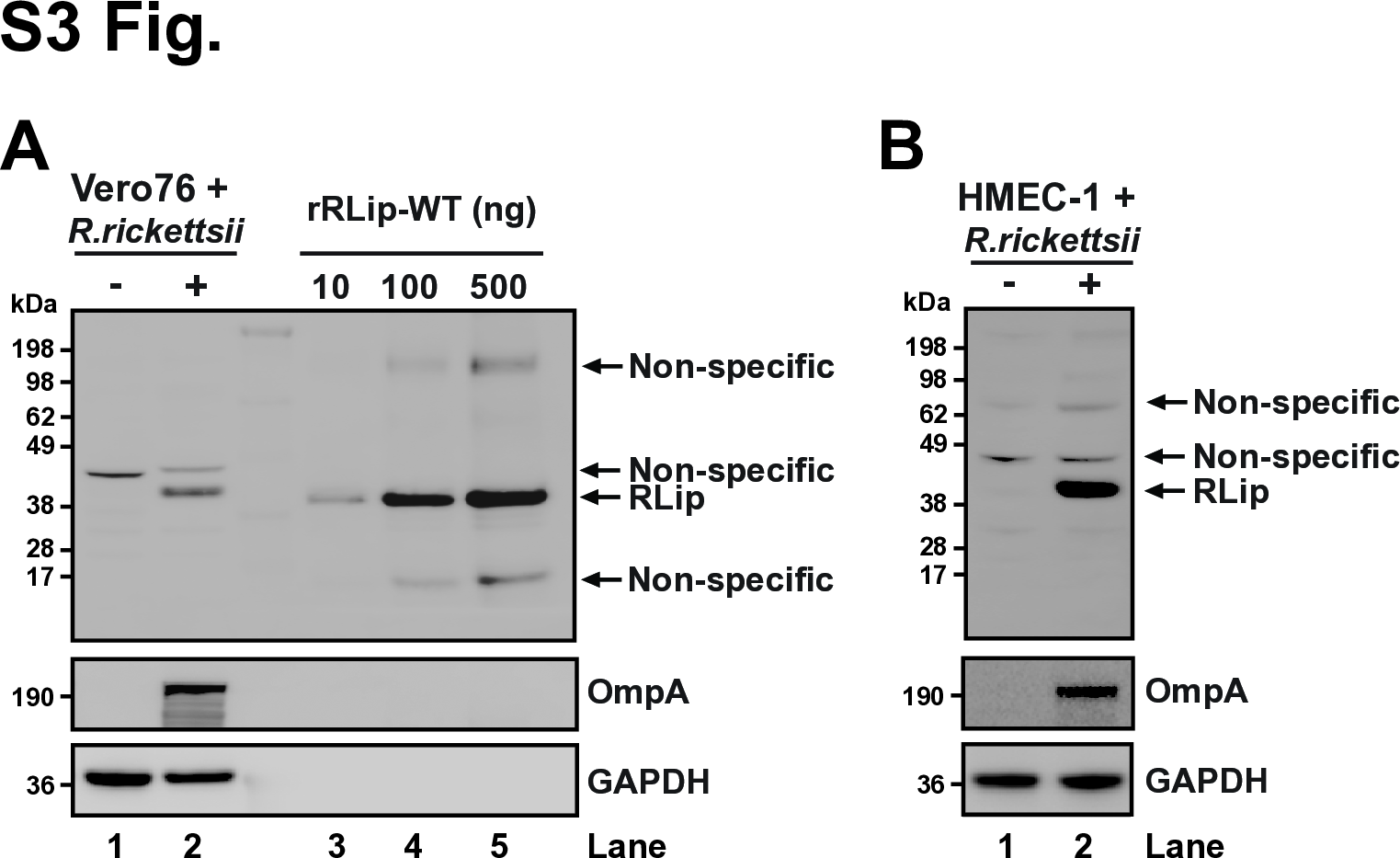

Supplement: S3 Fig — The recombinant RLip-WT protein, encoded by codon-optimized RLip gene (locus_tag: A1G_01170) was used to generate an anti-RLip Ab (RLip). The specificity of the anti-RLip Ab was validated by western blot analysis using whole cell lysates (WCL) of uninfected (lane 1) and R. rickettsii-infected (lane 2) Vero76 (A) or HMEC-1 cells (B). Purified recombinant (r)RLip-WT protein expressed in E. coli (A; lanes 3–5; 10–500 ng) was included as control. Immunoblotting with anti-OmpA/B and anti-GAPDH Abs was used to control for uninfected and R. rickettsii-infected cell lysates (A, and B; lanes 1 and 2). Images are a representative of 3 independent experiments. (TIF) [file pone.0332810.s003.tif]

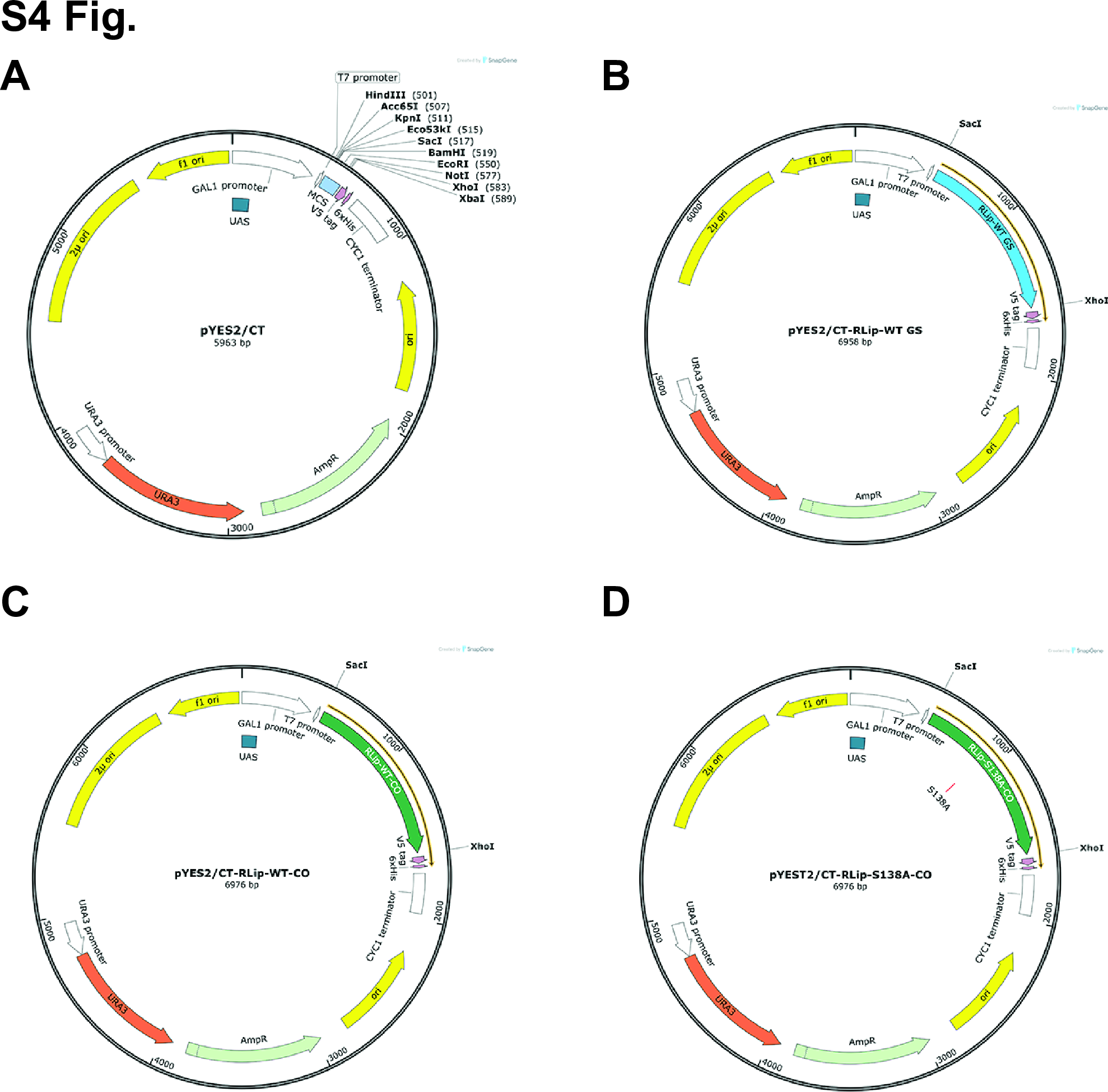

Supplement: S4 Fig — The empty yeast expression vector pYES2/CT with C-terminal epitope (V5 and 6x-His) tags (A), plasmid containing R. rickettsii genome sequence (GS) of the wild-type (WT) RLip gene (pYES2/CT-RLip-WTGS, B), and plasmids containing either codon-optimized (CO) gene encoding RLip-WT (pYES2/CT-RLip-WT-CO, C) or RLip-S138A-CO (pYES2/CT-RLip-S138A-CO, D) were used for transformation into S. cerevisiae strain INVSc-1, to perform the cytotoxicity assay. (TIF) [file pone.0332810.s004.tif]

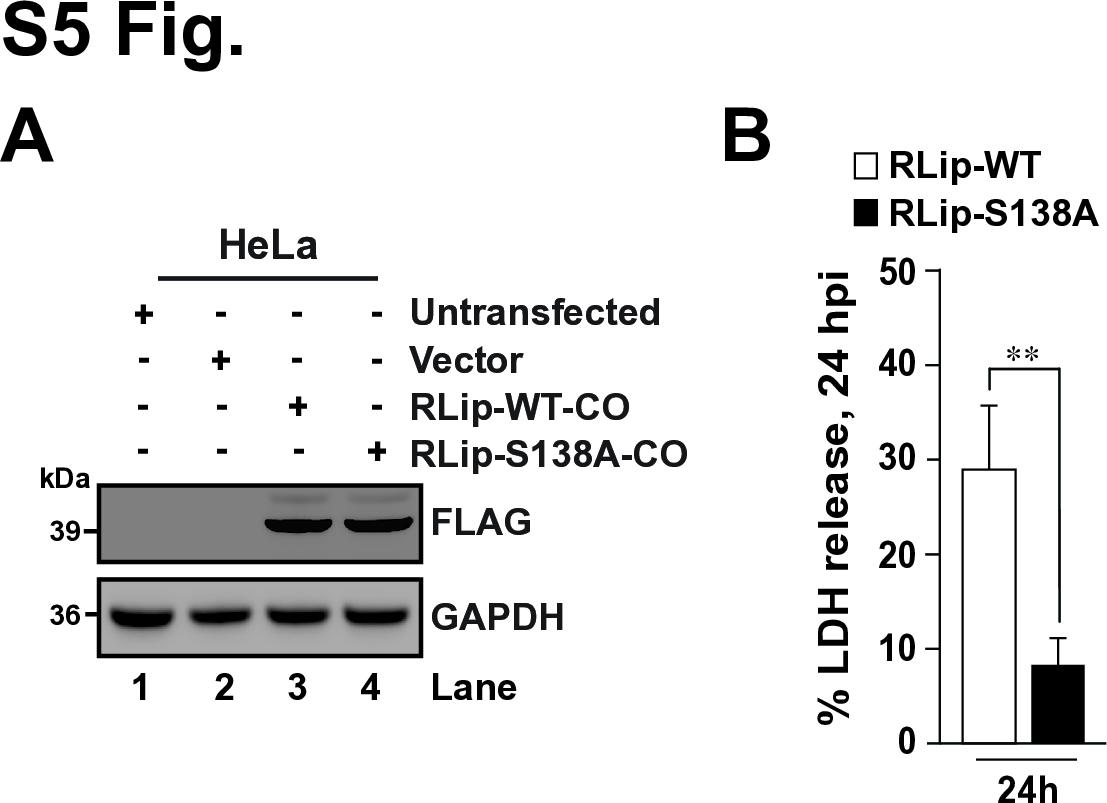

Supplement: S5 Fig — (A, B) Cellular cytotoxicity was evaluated in untransfected or HeLa cells transfected with pcDNA4-Flag empty vector, pcDNA4-Flag-RLip-WT, or pcDNA4-Flag-RLip-S138A. (A) Cell pellets were collected 24 hrs post-transfection, lysed, and analyzed for RLip-WT and RLip-S138A expression by immunoblotting using anti-Flag and anti-GAPDH Abs. (B) Supernatants from the same experiment were utilized to measure the cellular cytotoxicity by using lactate dehydrogenase (LDH) release assay following manufacturer’s instructions. Cytotoxicity levels from RLip-WT or RLip-S138A transfected cells were normalized by values from untransfected and empty vector transfected cells. Images shown in panel A is a representative of 3 independent experiments. Error bars in panel B represent means ± SEMs (standard errors of the means) from 4 independent experiments; **P ≤ 0.01. (TIF) [file pone.0332810.s005.tif]

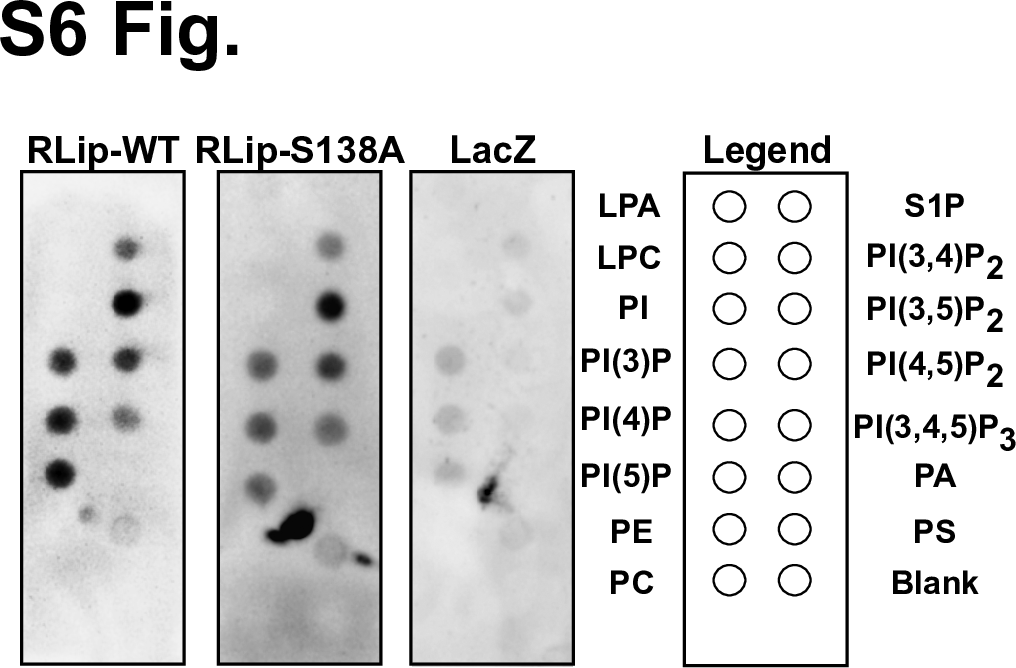

Supplement: S6 Fig — Lipid membrane assays (Echelon) were performed as per manufacturer’s instructions. The membrane was spotted with 1 μg of purified 6x-His-tagged rRLip-WT or rRLip-S138A protein and incubated for 1 h at room temperature. 6x-His-tagged rLacZ protein was used as a non-binding control. Binding of RLip to phosphoinositides was detected using an anti-His and HRP-conjugated Ab. The lipid membrane assay is a representative of 3 independent experiments. (TIF) [file pone.0332810.s006.tif]

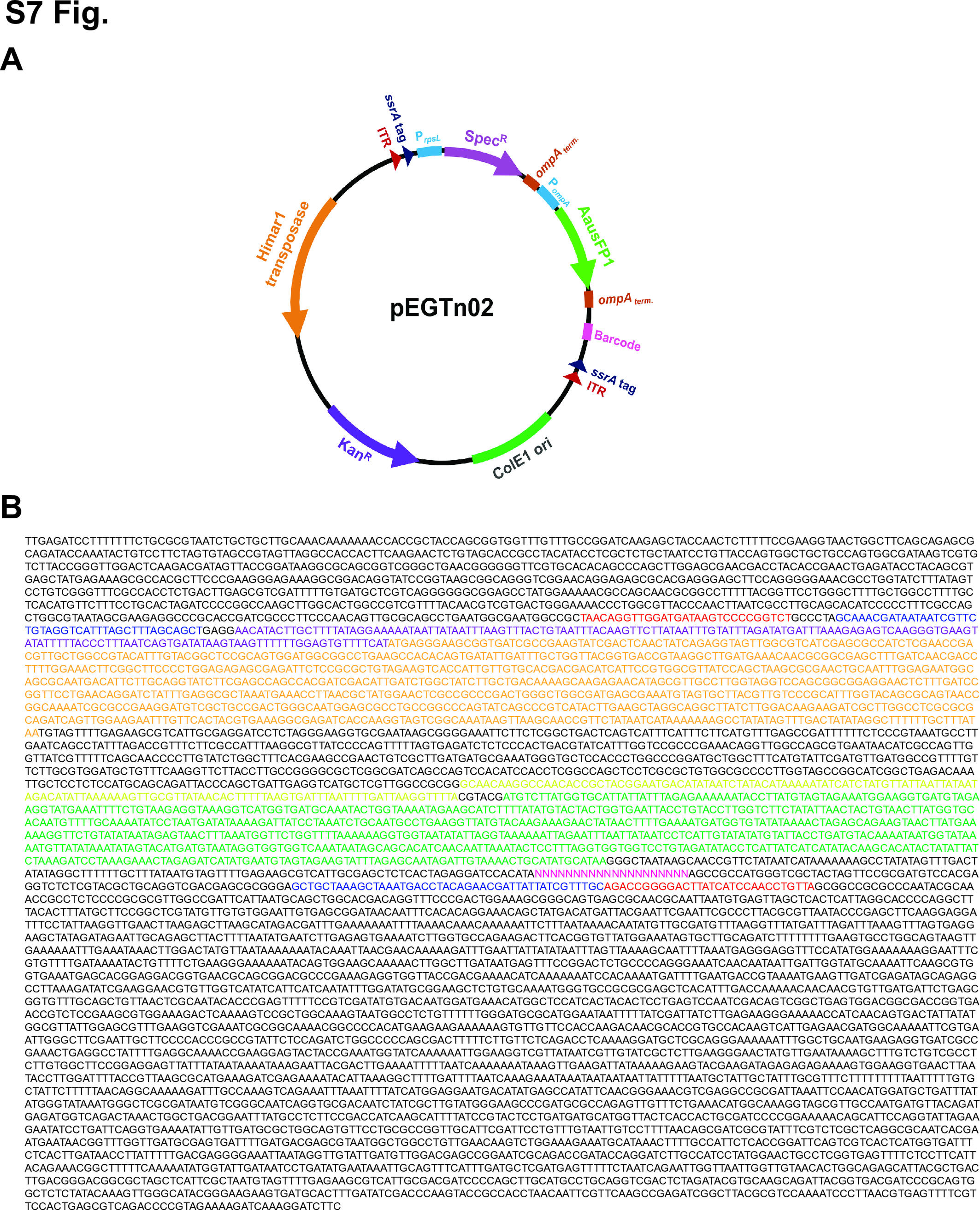

Supplement: S7 Fig — (A) Vector map of barcoded pEGTn02 plasmid used to generate the RLip transposon mutant. (B) Selected sequence information is highlighted by color: ITR, SsrA, PrpsL, specR, PompA, AausFP1, Barcode. (TIF) [file pone.0332810.s007.tif]

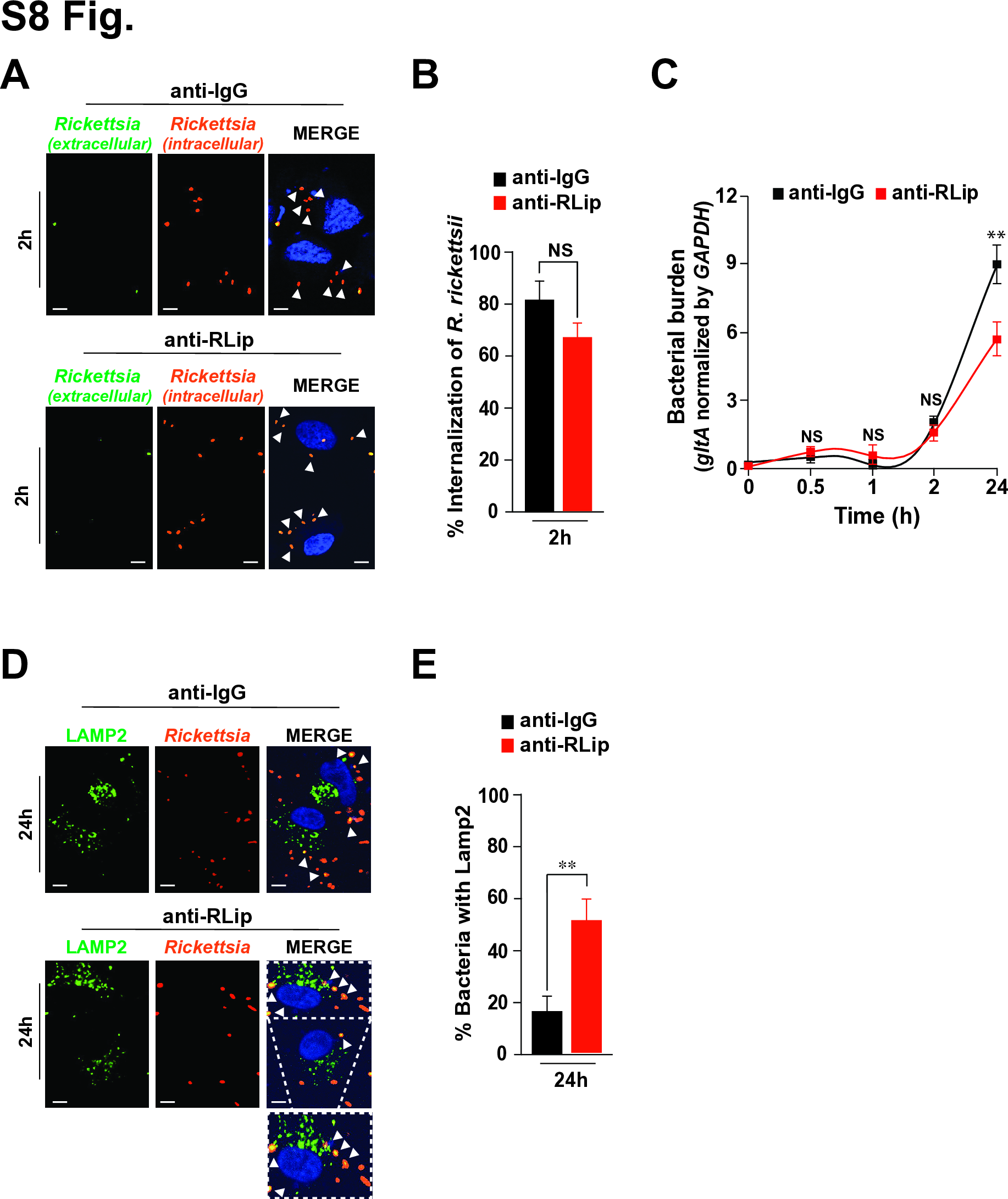

Supplement: S8 Fig — Partially purified R. rickettsii were pre-treated with 50 µg of affinity purified anti-RLip, or pre-immune IgG for 30 min on ice. Pretreated rickettsiae were added onto HMEC-1 monolayer and incubated for various length of time at 34°C and 5% CO2. (A, B) Extracellular and intracellular rickettsiae were assessed at 2 hpi by IFA using a MOI: 20 via differential staining using Alexa Fluor-488- and −594-conjugated anti-Rickettsia guinea pig serum as described in the Materials and Methods section. (C) Bacterial burden of antibody-treated rickettsiae in infected HMEC-1 cells was assessed for various length of time by rickettsial housekeeping citrate synthase (gltA) gene expression using RT-qPCR. GltA expression was normalized with respect to GAPDH transcription level as described in the Materials and Methods section. (D-E) Colocalization of antibody-treated R. rickettsii with LAMP2 was evaluated by IFA at 24 hpi using a MOI: 5 and Alexa Fluor-488-conjugated anti-LAMP2 Ab and Alexa Fluor-594-conjugated anti-Rickettsia guinea pig serum. Inset shows a close-up representation of Rickettsia-LAMP2 staining of HMEC-1 cells infected with anti-RLip Ab treated bacteria at 24 hpi. The cell nuclei were stained with 4’,6-diamidino-2-phenylindole (DAPI). Numbers of extracellular and intracellular rickettsiae (A-B) as well as colocalization between Rickettsia and LAMP2 (D-E) was analyzed using Coloc 2 plugin Fiji software. Bars in panels A, and D, 10 μm. Approximately 200 bacteria-infected cells were counted per condition and time point. Error bars (B, C, and E) represent means ± standard error of the mean (SEM) from 3 independent experiments; NS, not significant; **P ≤ 0.01. (TIF) [file pone.0332810.s008.tif]

**S10 Fig.**

**S3A Fig**

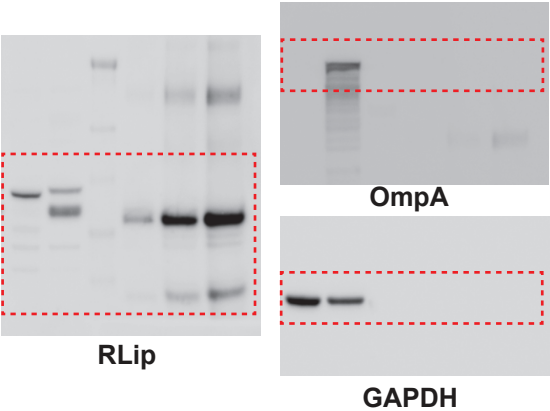

**S3B Fig**

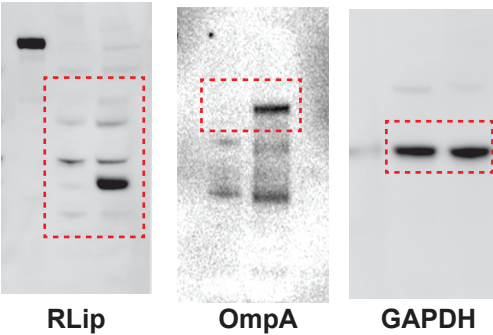

**S5A Fig**

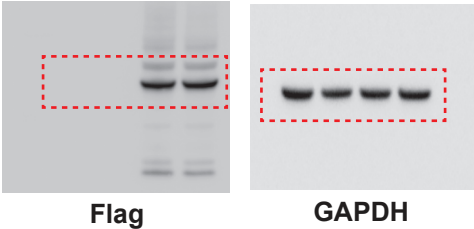

**red** highlighted area is the cropped image shown in the paper figures

Supplement: S10 Fig — S3A, B Fig: The recombinant RLip-WT protein was used to generate an anti-RLip Ab (RLip). The specificity of the anti-RLip Ab was validated by western blot analysis using whole cell lysates (WCL) of uninfected (lane 1) and R. rickettsii-infected (lane 2) Vero76 (A) or HMEC-1 cells (B). Purified recombinant (r)RLip-WT protein expressed in E. coli (A; lanes 3–5; 10–500 ng) was included as control. S5A Fig: Cellular cytotoxicity was evaluated in untransfected or HeLa cells transfected with pcDNA4-Flag empty vector, pcDNA4-Flag-RLip-WT, or pcDNA4-Flag-RLip-S138A. (A) Cell pellets were collected 24 hrs post-transfection, lysed, and analyzed for RLip-WT and RLip-S138A expression by immunoblotting using anti-Flag and anti-GAPDH Abs. Red highlighted area presents the cropped images shown in the supporting figures. (PDF) [file pone.0332810.s010.pdf]
