# Supplementary material for: Pathogenic rickettsiae encode a secreted lipase that facilitates intracytosolic colonization in host cells
Source: PLoS One. 2025 Oct 8;20(10):e0332810. doi: 10.1371/journal.pone.0332810 (PMC12507273; doi:10.1371/journal.pone.0332810)
Supplement: S9 Fig — Fig 1F, G: Uninfected or R. rickettsii-infected Vero76 (F) or HMEC-1 (G) cells were separated into cytoplasmic (C) and pellet (P) fractions. Samples were immunoblotted with anti-RLip, anti-Pat1, anti-OmpA/B, or anti-GAPDH Abs. Whole cell lysates (WCL) were used as expression control for all target proteins. Fig 1H: Partially purified rickettsiae were incubated with Vero76 cells for various length of time and whole host lysates were analyzed by immunoblotting as described in the Materials and Methods section. Fig 2A: HMEC-1 cells were infected with spotted fever group (SFG) rickettsiae, including R. rickettsii, R. parkeri, and R. montanensis for up to 48 hrs and analyzed by western blot analysis using anti-RLip, anti-Pat1, anti-OmpA/B, or anti-GAPDH Abs. Fig 4A, B: (A) Evaluation of the intact rlip gene and rlip transcript using R. parkeri WT or R. parkeri rlip::Tn bacterial DNA. (B) RLip protein expression was detected in HMEC-1 cells infected with R. parkeri WT or R. parkeri rlip::Tn bacteria by western blot analysis using anti-RLip, and anti-GAPDH Abs. Red highlighted area presents the cropped images shown in the main figures. (PDF) [file pone.0332810.s009.pdf]

S9 Fig.

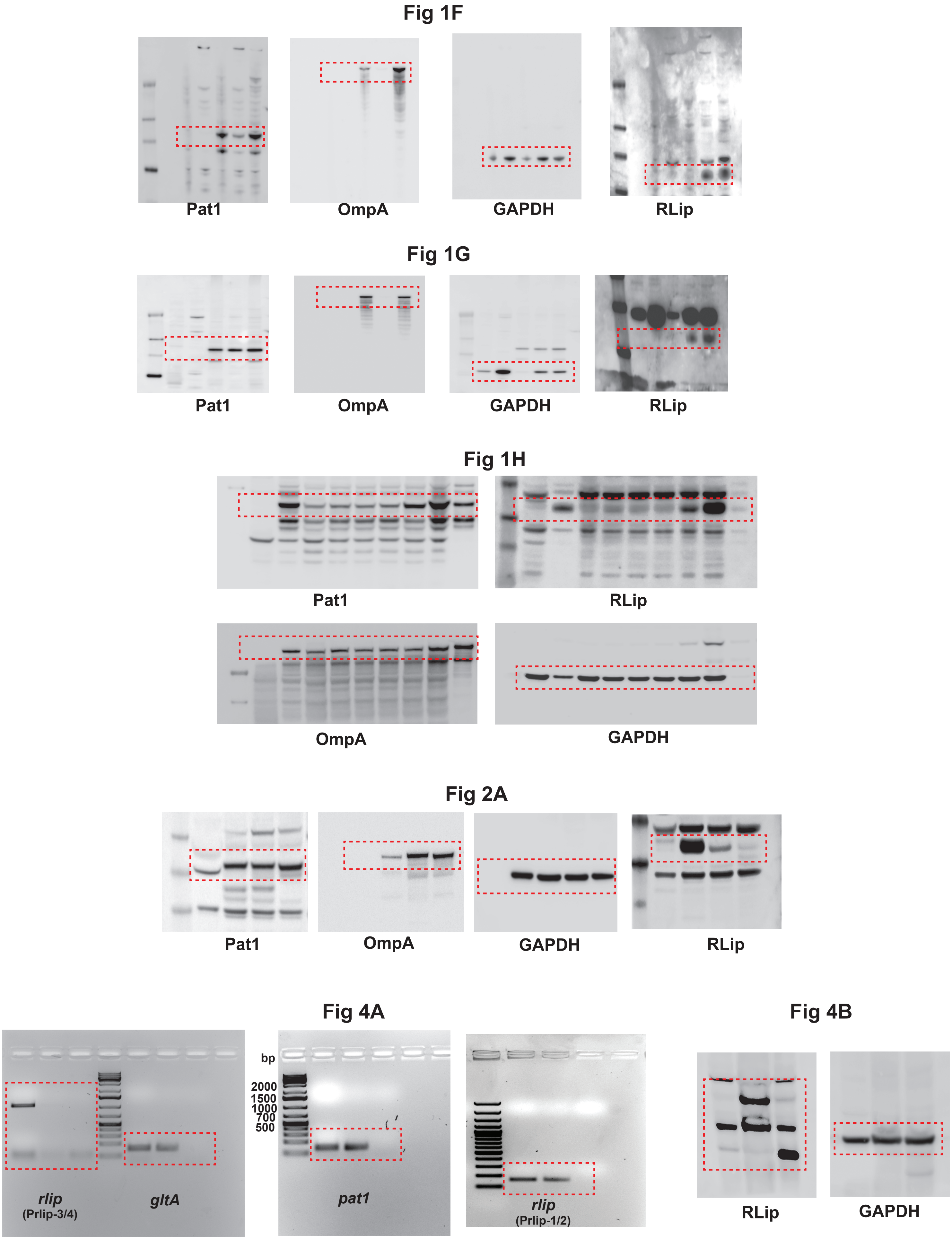

red highlighted area is the cropped image shown in the paper figures
